# Dynamic frustrated charge hotspots created by charge density modulation sequester globular proteins into complex coacervates†

**DOI:** 10.1039/d3sc00993a

**Published:** 2023-05-19

**Authors:** Biplab K C, Teruki Nii, Takeshi Mori, Yoshiki Katayama, Akihiro Kishimura

**Affiliations:** a Department of Applied Chemistry, Faculty of Engineering, Kyushu University 744 Moto-oka, Nishi-ku Fukuoka 819-0395 Japan kishimura.akihiro.776@m.kyushu-u.ac.jp; b Graduate School of Systems Life Sciences, Kyushu University 744 Moto-oka, Nishi-ku Fukuoka 819-0395 Japan; c Center for Future Chemistry, Kyushu University 744 Moto-oka, Nishi-ku Fukuoka 819-0395 Japan; d Center for Molecular Systems, Kyushu University 744 Moto-oka, Nishi-ku Fukuoka 819-0395 Japan; e Center for Advanced Medical Open Innovation, Kyushu University 3-1-1 Maidashi, Higashi-ku Fukuoka 812-8582 Japan; f Department of Biomedical Engineering, Chung Yuan Christian University 200 Chung Pei Rd. Chung Li Taiwan 32023 ROC; g RIKEN Center for Emergent Matter Science 2-1 Hirosawa, Wako Saitama 351-0198 Japan

## Abstract

This study presents a simple strategy for the sequestration of globular proteins as clients into synthetic polypeptide-based complex coacervates as a scaffold, thereby recapitulating the scaffold-client interaction found in biological condensates. Considering the low net charges of scaffold proteins participating in biological condensates, the linear charge density (*σ*) on the polyanion, polyethylene glycol-*b*-poly(aspartic acids), was reduced by introducing hydroxypropyl or butyl moieties as a charge-neutral pendant group. Complex coacervate prepared from the series of reduced-*σ* polyanions and the polycation, homo-poly-l-lysine, could act as a scaffold that sequestered various globular proteins with high encapsulation efficiency (>80%), which sometimes involved further agglomerations in the coacervates. The sequestration of proteins was basically driven by electrostatic interaction, and therefore depended on the ionic strength and charges of the proteins. However, based on the results of polymer partitioning in the coacervate in the presence or absence of proteins, charge ratios between cationic and anionic polymers were maintained at the charge ratio of unity. Therefore, the origin of the electrostatic interaction with proteins is considered to be dynamic frustrated charges in the complex coacervates created by non-neutralized charges on polymer chains. Furthermore, fluorescence recovery after photobleaching (FRAP) measurements showed that the interaction of side-chains and proteins changed the dynamic property of coacervates. It also suggested that the physical properties of the condensate are tunable before and after the sequestration of globular proteins. The present rational design approach of the scaffold-client interaction is helpful for basic life-science research and the applied frontier of artificial organelles.

## Abstract 2

Reducing the charge density of linear synthetic polyion creates frustrated hotspots in complex coacervates, which allow the sequestration of globular proteins electrostatically mimicking “scaffold-client” interaction of biological condensate.

## Graphical abstract

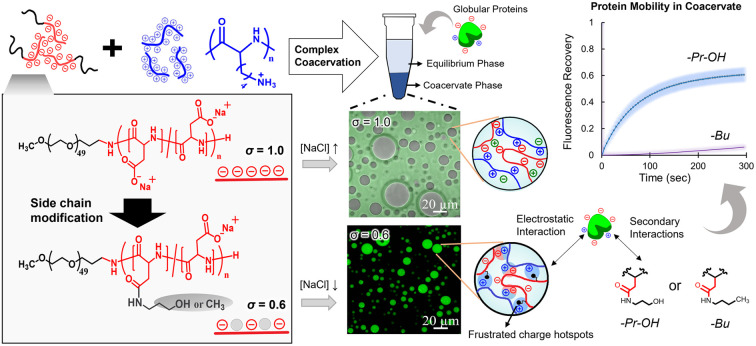

## Introduction

Complex coacervation is one of the aqueous liquid–liquid phase separation (LLPS) processes driven by mixing two or more aqueous solutions of oppositely charged materials, which involves the formation of a condensed phase. The resulting condensed phase is usually called a coacervate as an associative complex formed *via* electrostatic interaction.^1^ Such coacervate phases have unique properties like low surface tension,^2^ low dielectrics, high viscosity, and a macromolecular crowding environment, which can provide a unique compartmentalized microenvironment. Synthetic-polymer-based complex coacervates have already been used for many applications including microencapsulation^3^ and underwater adhesives.^4^ In the case of natural systems, recent studies have reported numerous kinds of biomolecular condensates having distinct biological functions and roles in pathophysiology, which are formed *via* LLPS.^5–7^ In most cases, the biomolecular condensate formation is driven by complex coacervation, and functions in the cytosol without a lipid membrane.^8^ Because LLPS is the physical process, the biological functions of these condensates, such as biomolecule partitioning, activation and inhibition of reactions, and hierarchical organization, depend on the physical and thermodynamic parameters of the condensates, *e.g.*, surface tension, viscosity, and chemical potentials. These parameters are governed by interactions among constituting biomacromolecules of the condensate. By mimicking the molecular design and interaction of the components of biomolecular condensate, synthetic-polymer-based complex coacervates can easily recapitulate such parameters in artificial systems. Hence, it can give optimal models for elucidating the relationship between molecular interactions and biological functions.^9–11^ Thus, such systems have been used as an artificial model for biological condensate.

One of the key features of biomolecular condensate is that they work as a “scaffold” to recruit numerous functional globular proteins as “clients,” which do not necessarily partake in the LLPS process but are critically associated with the biological functions of the condensate.^12^ For example, for the formation of cellular bodies, more than a hundred different proteins are recruited as clients, and only a few different types of proteins act as a scaffold.^13^ Many studies have identified key proteins that can reconstitute the scaffold of the condensate *in vitro*, particularly based on the recombinant protein-based approaches.^12,14^ Such scaffold proteins are usually intrinsically disordered proteins (IDPs) or contain intrinsically disordered regions (IDRs), which are characterized by a high conformational degree of freedom, and, in many cases, multivalent interaction sites.^12,15^ Because such IDPs/IDRs do not follow a classical sequence-structure–function relationship, the behaviour of the IDPs depends on the sequence-ensemble average values of coarse-grained parameters of the sequence, such as charge density, number of charges, and hydropathy.^16^ Hence, one of the key challenges is to relate such parameters with biological functions, such as client partitioning. Although recombinant-protein-based *in vitro* experiments have succeeded in decoding some of the key relations among IDPs, condensates, and biological functions,^17–19^ synthetic polypeptides can provide a new approach to tackling the issue from the viewpoint of macromolecular sciences. Particularly, the design flexibility of synthetic polypeptides can be a powerful tool, which allows fine-tuning of physical and chemical parameters, *e.g.*, charge density, hydrophobicity, hydrogen bonding capability, and so on. Following advancement in the synthesis and modification methods,^20,21^ properties of the synthetic polypeptides can now be tuned to have to determine the correlations that are difficult to access *via in vivo* studies. This can circumvent the complexity associated with numerous biomacromolecules in natural cytosolic conditions, which are the main hindrances in developing general molecular principles. Therefore, various theoretical and computational models in synthetic-polymer-based complex coacervates have recently been updated, considering subtle variables, such as the total charge number, charge density, charge patterning, peptide length, and hydrophobicity in compliance with the biological system.^22–26^ However, wet-lab research has been limited in terms of exploring these subtle variables and their relationship with functions of the condensates.

One major discrepancy between synthetic and natural system is that the chemical properties of polypeptides vary considerably from those of IDPs/IDRs that drive intracellular phase separation. Approximately 70% of these IDPs have low charge density,^16^ defined as net charge per residue, whereas most synthetic polypeptides have high charge density. Linear charge density on polymer chains is essential to regulating phase separation.^26^ Even in *in vivo* systems, phosphorylation and acetylation found in post-translational modifications regulate LLPS by modulating the charge density of IDPs/IDRs.^27–29^ Hence, the understanding of the effect of the charge density on the physio–chemical properties of the scaffold and on client partitioning and dynamics is critical in the biological context. Recent studies have focused on elucidating the protein sequestration behaviour in the complex coacervate, considering protein and polymer properties.^30–33^ One of the interesting findings is the significance of charge patches of proteins, isoelectric point, and the effect of pH on the partitioning of proteins into coacervate, which provides some insight into molecular interactions between globular proteins and polyelectrolytes.^31^ Herein, we took a different approach to study the effect of charge density of the polypeptides by modifying sidechain functionalities of polyelectrolytes and to clarify the encapsulation behaviour of various proteins into coacervates. We first designed a synthetic version of the “scaffold-client” model *via* complex coacervation of synthetic-polypeptide-based polyions, in which the linear charge density (*σ*) of polyanions was reduced. Subsequently, behaviours of the sequestration of globular proteins (clients) were evaluated for the resulting coacervates. Finally, we attempted to elucidate the effect of the microenvironment modulated by side-chain functionalities on the dynamic nature of coacervates and sequestered proteins. This study allows for the recapitulation of the essence of a scaffold-client model in a synthetic system, where chemical and physical properties are predictable.

## Results and discussion

### Coacervation of charge reduced polyanions

**Fig. 1:**
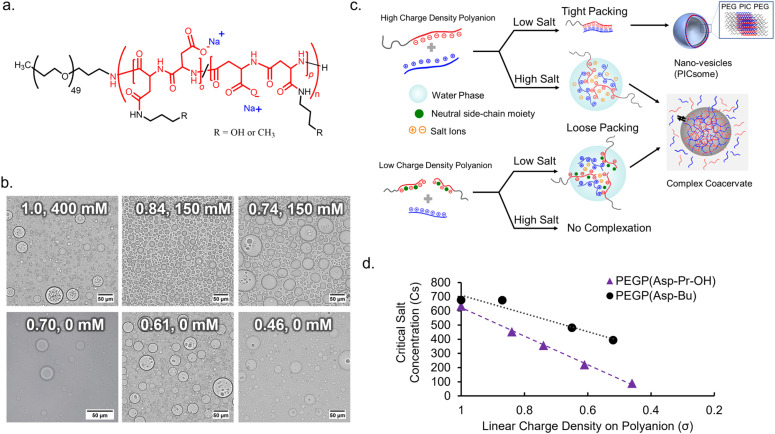
Coacervation behaviours of poly-(l-aspartate) (PAsp) with side-chain modifications. (a) Chemical structures of PAsp with pendant hydroxypropyl (Pr-OH) or butyl (Bu) groups. (b) Optical microscopic images of coacervate droplets prepared from polyanions with different charge density (*σ*) at the optimal NaCl concentration (*σ*, [NaCl]) (scalebar 50 μm). (c) Schematic diagram of effect of lowering charge density (*σ*) on coacervation. (d) Critical salt concentration for dissociation (Cs_dis_) of coacervates with different *σ*.

To obtain an appropriate synthetic scaffold, polypeptide-based complex coacervate was prepared from homo poly-l-lysine (PLL) and PEG-*b*-poly(α,β-aspartic acid) (PEG-PAsp) as cationic and anionic polyelectrolytes, respectively (see ESI† for details on synthesis methods). PEGylated block copolymer was used as a polyanion to enhance the resulting volume of coacervates compared to homo polyanion (data not shown). In addition, we expect PEG chains to function as a flexible moiety behaving similarly to the non-charged glycine/serine-rich regions found in IDPs, which can provide more conformational degrees of freedom within the coacervate.^34^ The linear charge density (*σ*) on the polyion, defined by the ratio of the number of charges per average degree of polymerization, was modulated *via* the random side-chain modification of the polymer with charge-neutral small groups. The random modification allows for charge heterogeneity throughout the sequence, which makes *σ* statistical the ensemble average. Simple side-chain modifications were selected for tuning *σ* to systematically observe the effect of electrostatic interactions. This was achieved *via* post-polymerization modification of precursor polymer, PEG-*b*-poly-(*o*-benzyl-l-aspartate) (PEG-PBLA), *via* partial aminolysis by 3-amino-propan-1-ol or *n*-butyl amine to varying degrees, followed by alkaline hydrolysis with NaOH_aq_. This gives the polymer possessing non-charged propanol (Pr-OH), PEG-P(Asp-Pr-OH), or possessing butyl (Bu) moieties, PEG-P(Asp-Bu), respectively as a pendant group (Fig. 1a and Schemes S2 and S3 in the ESI†). The degrees of modification were computed from ^1^H NMR. A higher degree of modification results in the decrease of *σ* (Table 1).

**Table 1:** Characterization of polyanions for varying *σ*

%-Modification	Degree of polymerization^a^	No. of modified residues per chain (*m*)	Linear charge density (*σ*)	*f*_PEG_ (%)
0%	82	0	1.0	9.6

**PEG-P(Asp-Pr-OH)**				
16%	83	13	0.84	10.4
26%	77	20	0.74	11.4
31%	78	24	0.69	11.5
39%	74	29	0.61	12.3
54%	85	46	0.46	11.4

**PEG-P(Asp-Bu)**				
13%	77	10	0.87	11.0
30%	82	25	0.70	11.0
35%	82	29	0.65	11.8
48%	84	40	0.52	11.3

a Degree of polymerization determined by ^1^H NMR (400 MHz).

Complex coacervate were prepared by simple pipette mixing PLL (average degree of polymerization (DP) of 110) and a series of polyanions at a cation-to-anion charge ratio of unity (cations : anions (C : A) = 1 : 1), in a 25 mM HEPES buffer (pH 7.4, various NaCl concentrations). The ionic strength of the system plays a critical role in effective coacervation given that the electrostatic interaction in the dielectric solvent depends on the charge screening from counterions. Therefore, for different charge density polyions, different NaCl concentration was required for effective coacervation. To obtain macroscopic phase separation state, or micrometer-sized droplets, from PEGlyated block copolymers, a volume fraction of PEG (*f*_PEG_) throughout the assembly should be regulated in the proper range (*f*_PEG_ < 10%) to avoid the formation of nanometer-scaled polyion-complex (PIC) assemblies.^35^ Therefore, DLS measurement and transmission electron microscopy (TEM) measurements were conducted for supernatants to confirm whether nano-assemblies were formed or not. Then, optimal NaCl concentration for coacervation was examined as a minimum NaCl concentration required for macroscopic phase separation without involving nano-assembly formation.

In the case of the polymer with *σ* < 0.7, micrometer-sized droplet formation occurred without NaCl despite having higher *f*_PEG_ (Fig. 1b). Conversely, for *σ* > 0.7, micrometer-sized droplet formation occurred only at relatively high ionic strength ([NaCl] ≥ 150 mM, Fig. 1b). Moreover, for *σ* > 0.7, reduced ionic strength triggered the formation of nano-assemblies (vesicles) confirmed *via* both DLS and TEM (ESI Fig. S15†). This suggests that charge screening from NaCl is essential for micrometer-sized droplet formation. Optimal NaCl concentrations were determined based on low count rate in DLS measurement of supernatant (ESI Fig. S16†). Presumably, more hydration of the PIC domain increases PIC volume to reduce values of actual *f*_PEG_ (Fig. 1c). The micrometer-sized droplet formation from polyanions with reduced *σ* (<0.7) at low NaCl concentrations can be explained by the lower density of polyion pairs contained in the PICs (ESI Fig. S17a†). The density of polyion pairs is correlated with the magnitude of interaction among the polymers, and its sensitivity to ionic strength change can be approximately interpreted as an inverse to *σ* (Table 1). Therefore, relatively low *σ* can contribute to lowering the threshold value of NaCl concentration for micrometer-sized droplet formation; we decided to use the polyanions with *σ* ≤ 0.87 for further protein sequestration experiments (*vide infra*).

**Fig. 2:**
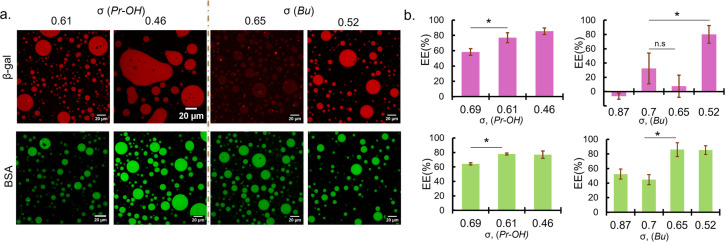
Protein sequestration behaviours of coacervates with reduced *σ*. (a) Fluorescence images of rhodamine-labelled β-gal (red) and FITC labelled BSA (green) sequestered in coacervate droplets prepared at 0 mM NaCl (left, PEG-P(Asp-Pr-OH)-based coacervate; right, PEG-P(Asp-Bu)-based coacervate). The values represent the linear charge density of polyanions (*σ*). (b) Encapsulation efficiency (EE) of β-gal (top) and BSA (bottom) for coacervates with different *σ* (*n* = 3, * = *p* < 0.05. n. s: not significant).

It is also possible to define critical salt concentration for dissociation (Cs_dis_), where coacervate microdroplets are dissolved and dissociated (ESI Fig. S17b†). Cs_dis_ was defined as the NaCl concentration at the point of inflection of the turbidity curve plotted against increasing salt concentration (ESI Fig. S17c and d†). Cs_dis_ decreased linearly with decreasing *σ* for both PEG-P(Asp-Pr-OH) and PEG-P(Asp-Bu) (Fig. 1d). This trend is reasonable because *σ* reflects the density of ion-pairs as described above. In fact, the Cs_dis_ value depends primarily on two factors – number of ion-pairs between polyions and strength of each ion-pair.^36^ Furthermore, the difference between the slopes of regression lines for two sample series suggests that PEG-P(Asp-Bu)-based coacervates are less sensitive to salt concentration change (Fig. 2b). This result is consistent with the concept that the interaction strength of each ion-pair is dependent on its surrounding microenvironment. Butyl-modified polyanions, PEG-P(Asp-Bu), can provide a more hydrophobic environment, which strengthens the electrostatic interaction compared to that for PEG-P(Asp-Pr-OH). The refractive index (RI) measurement of coacervate microdroplets using the holographic visual microscopy technique showed higher RI value for PEG-P(Asp-Bu)-based coacervates (1.403 ± 0.03) compared to that of PEG-P(Asp-Pr-OH)-based coacervates (1.391 ± 0.005) (ESI Fig. S18†), supporting difference in the dielectric constants of coacervates with different functionalities.^37–39^

### Protein sequestration in complex coacervates

To establish the synthetic model system, where globular proteins act as clients and do not contribute to LLPS, protein sequestration into *σ*-reduced polyanion-based droplets was investigated. Bovine serum albumin (BSA; pI 5.2 and MW 66.5 kDa) and β-galactosidase (β-gal; pI 4.6 and MW 540 kDa) were used as model proteins, because they are negatively charged at pH 7.4 and have different molecular weights (ESI Table S3†). In the presence of fluorescence-dye labelled proteins, PLL and *σ*-reduced polyanions were mixed at C : A = 1 : 1 in 25 mM HEPES buffer (pH 7.4, 0 mM NaCl). Charges from the proteins were not considered upon preparation because the protein amount was trivial compared to the polymer amount (typically 6.25 μg of protein and 194–285 μg of polymers at preparation). In fact, the contribution of charge from the proteins (charge per mass ratio) used in this study are very low compared to that of polyions (ESI Table S4†).

Sequestration of protein was verified by fluorescence microscopy observation and encapsulation efficiency was calculated from the fluorescence intensity measurement of the dilute phase. Fluorescence images showed that BSA were favourably sequestered into the coacervates prepared at the low ionic strength condition (0 mM NaCl) for *σ* < 0.7 regardless of the functionalities (Fig. 2a). The encapsulation efficiency of the coacervates also increases with decreasing *σ*, reaching a maximum of approximately 80% (Fig. 2b). Given the small volume of coacervate (∼2 μL) compared to the total sample volume (120 μL), this is at least a 50-fold increase in concentration. In the case of β-gal, the encapsulation trend is similar for PEG-P(Asp-Pr-OH)-based coacervates. However, at relatively high *σ* (0.6–0.8), PEG-P(Asp-Bu)-based coacervates favoured BSA but not β-gal for encapsulation (Fig. 2). This disparity between BSA and β-gal for different functional groups can be attributed to the surface property difference between two proteins and their interactions with the coacervate microenvironment. Although both proteins are negatively charged, their surface-charge distribution and other parameters like size and hydrophilicity differ. Furthermore, properties of coacervate microenvironment, especially a dielectric constant, would be greatly affected by side-chain functionalities, thus affecting the protein partitioning. However, higher encapsulation of both proteins at lower *σ* (∼0.5) regardless of the functionalities suggest that the strategy of charge density reduction can be applied for many types of protein. Fluorescein-labelled β-gal and Cy5-labelled BSA also showed similar profiles for protein encapsulation, suggesting results are independent of labelling dyes (ESI Fig. S19†). Interestingly, the proteins were sequestered by the ready-made coacervates, indicating the coacervate can work as a sponge for absorbing proteins from the surrounding (ESI Movies S1, 2 and 3†). This behaviour is consistent with the scaffold-client model found in biological systems where client proteins are partitioned into a biomolecular condensate, depending on a multi-valency of scaffold molecules (usually IDPs/IDRs), which provide multiple interaction sites and possess flexible main-chain conformation. Such scaffold molecules can function as a main regulator and driver of the condensate formation.^5^ Notably, at 50 mM NaCl, PEG-P(Asp-Bu) with *σ* = 0.7 and 1.0 gave coacervates, and only the coacervate with *σ* = 0.7 showed successful encapsulation of FITC-BSA, validating our strategy of charge density reduction (ESI Fig. S20†). Moreover, for polyanions with *σ* > 0.7, higher ionic strength (≥150 mM) was required for coacervation, but we found no encapsulation of both proteins (ESI Fig. S21†).

**Fig. 3:**
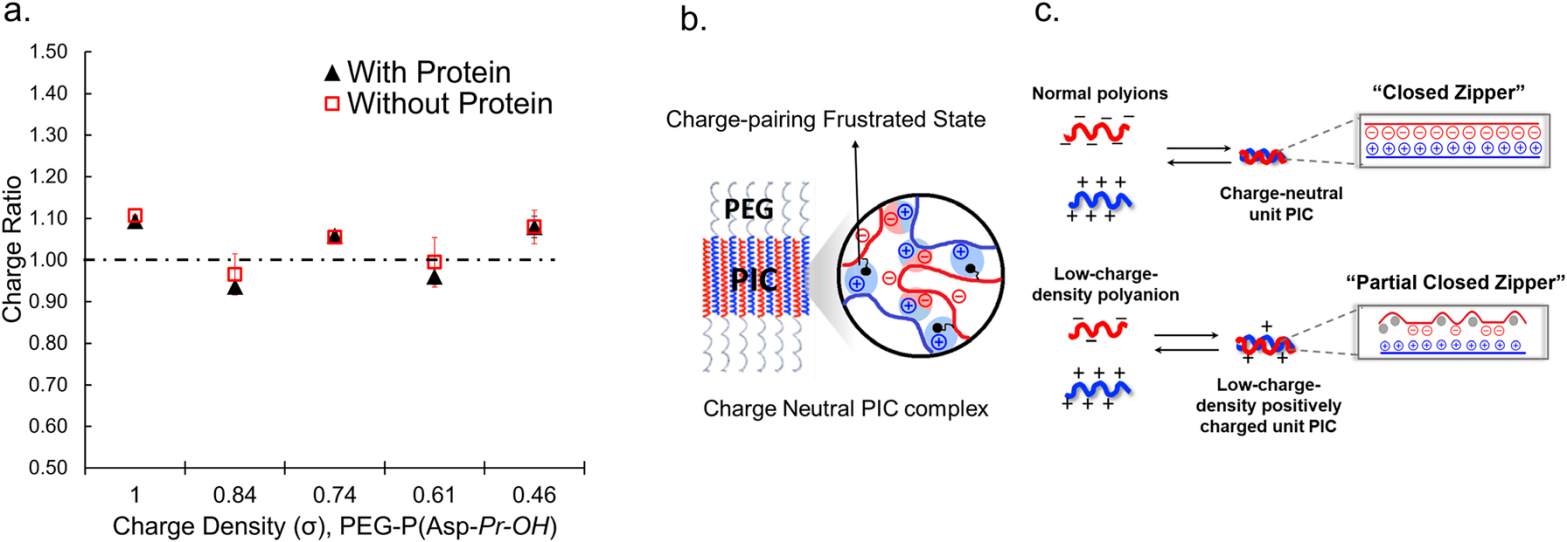
Mechanistic consideration of the polyion complexation in *σ*-reduced coacervates. (a) Charge ratios of polyions within the coacervates with different *σ* determined from the polymer partitioning in the presence and absence of protein, β-gal. (b) A schematic diagram of frustrated charge hotspots within a polyion complex (PIC) domain. (c) A unit PIC model for two types of PICs showing charge-balanced and imbalanced states within the PIC. A closed zipper model (top) and partial closed zipper model (bottom).

**Fig. 4:**
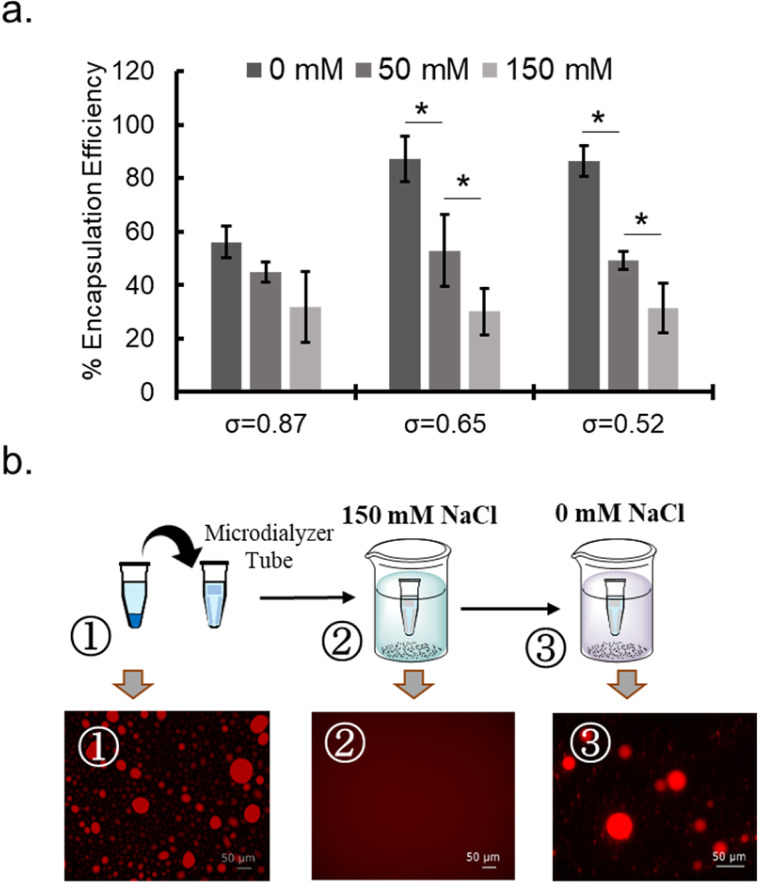
NaCl sensitivity of coacervates. (a) NaCl-concentration dependency of the encapsulation efficiency (%) of BSA for PEG-P(Asp-Bu)-coacervates with different *σ*. (b) Sequestration and release behaviour of rho-β-gal for PEG-P(Asp-Pr-OH)-based coacervate where *σ* = 0.46 in response to the NaCl concentration change between high (150 mM) and low (0 mM) conditions.

When proteins were sequestered *via* electrostatic interaction by the coacervate prepared from charge density-reduced polypeptides, coacervate droplets appeared to yield net non-neutralized charges. To determine the origin of the net charges, the actual composition of the coacervate was evaluated. For that, both polyions were labelled with fluorescence dyes, and the partitioning of polymers in dilute and coacervate phase was evaluated separately (ESI Fig. S22†). The charge ratio calculated from actual polymer partitioning was almost 1 : 1 and did not differ greatly for modified and non-modified coacervate regardless of the presence or absence of protein (β-gal) prepared at optimum NaCl concentration for coacervation (Fig. 3a). This result implies charge neutrality of the coacervate phase. Furthermore, proteins do not affect the polyion partitioning in coacervate. The coacervate would simply act as a solvent, where proteins are accommodated as a client. The charged nature of the coacervate might be the result of fluctuation at the local microenvironment level, where imbalanced charge can appear as a transient and dynamic frustrated state, because of the inability of the *σ*-reduced polyions to completely cancel the charges of the counter-part polyelectrolytes (PLL in the present study) in a zipper-closing manner (Fig. 1c and 4b). In other words, charge-neutral side chains facilitate the formation of hotspots of non-neutralized charges temporally, which makes the coacervate compatible with charged proteins *via* electrostatic interaction (Fig. 3b). Furthermore, when the protein concentration (FITC-BSA) increased up to 10 mg mL^−1^, protein encapsulation efficiency remained almost constant at ∼80% (ESI Fig. S23a†). This result indicates that our coacervate can act as a solvent in the protein sequestration, which is presumably governed by partitioning coefficient. This idea is consistent with the decrease of polyion to protein ratios in coacervates in accordance with the increase in BSA concentration (ESI Fig. S23b†).

To probe the nature of interaction between protein and polymer, NaCl concentration dependency of protein sequestration was investigated. The increase in NaCl concentration inhibited protein sequestration into the coacervate (Fig. 4a and ESI S24a†). In addition, when the NaCl concentration was gradually increased in the sample of protein-sequestered coacervates, proteins were released from the coacervate phase (ESI Fig. S24b†). As expected, PEG-P(Asp-Pr-OH) coacervate was more sensitive to the increase in concentration of NaCl compared to that of PEG-P(Asp-Bu) coacervate for similar *σ* values. More hydrophobic butyl-modification can reduce the dielectric constant of the droplet, which can partly compensate for the reduced electrostatic interaction in the presence of NaCl. Notably, the response to NaCl concentration change was reversible, and was verified in the cyclic encapsulation and release experiments upon dialysis against low and high salt buffer conditions (Fig. 4b). Furthermore, SEC measurements demonstrated the release of protein under the higher NaCl concentration concentrations happened not as a PIC form but as a liberated form from the charged polymers (ESI Fig. S25†). Therefore, sequestration of proteins can be basically driven by electrostatic interaction.

Recent *in vivo* studies have proposed the use of hexane-1,6-diol as the probe for weak hydrophobic interactions in droplets.^40^ However, treatment with hexane-1,6-diol up to 20 wt% had no significant effect on the protein sequestration in this study (ESI Fig. S26†). Therefore, weak hydrophobic interaction is not one of the main drivers for protein sequestration, but can make minor contributions, such as sensitivity to ionic strength change and protein sequestration trend, as the effect of side-chain functionalities.

Notably, proteins formed agglomerations (granules) after sequestration into droplets over prolonged overnight incubation for both PEG-P(Asp-Pr-OH) and PEG-P(Asp-Bu)-based coacervates. However, the agglomeration behaviour was more prominent with β-gal than with BSA (ESI Fig. S27†). Because the agglomeration was promoted for prolonged incubation time and relatively high temperature (ESI Fig. S28†), and dissolved at the higher NaCl concentration (ESI Fig. S24b†), this protein agglomeration is not a transient state and can be regarded as a thermodynamically favoured state. Furthermore, the increment of incubation temperature facilitated the agglomeration, implying that the process is entropy-driven. Collectively, a slight difference in the coacervate microenvironment and protein nature can result in further phase separation of proteins within droplets. Because one of the key mechanisms of pathological amyloid formation within the cell can be traced to the hardening of the condensate sequestrating proteins,^41,42^ it was interesting to see if our model of β-gal could be used to probe such a mechanism. This might provide helpful insight for proper therapeutic intervention of such amyloid driven pathology as ALS and Alzheimer's disease.

To elucidate the properties of protein suitable for effective sequestration, six fluorescent and fluorescence-labelled proteins: *viz.* EGFP, lysozyme, IgG, glucose oxidase (GOx), horse radish peroxidase (HRP), diamine oxidase (DaO) different molecular weights and charges were tested for encapsulation (ESI Table S3†). Our findings suggest that, as the *σ* of polyanions decreases, most proteins can be positively partitioned within the coacervates (ESI Fig. S29 and S30†). For proteins with a relatively low number of total charges, lysozyme, IgG, and HRP, the partitioning was lower than that of proteins with a relatively high number of total charges, EGFP, GOx and DaO, where sufficient electrostatic interactions between proteins and poly-ions can occur. However, there were some exceptions like lysozyme could be loaded at high butyl modification (*σ* = 0.52), which might be the result of specific protein properties. Therefore, the contribution of protein properties, such as surface charge distribution pattern and hydrophobicity, needs to be considered in further investigation.

**Scheme 1:**
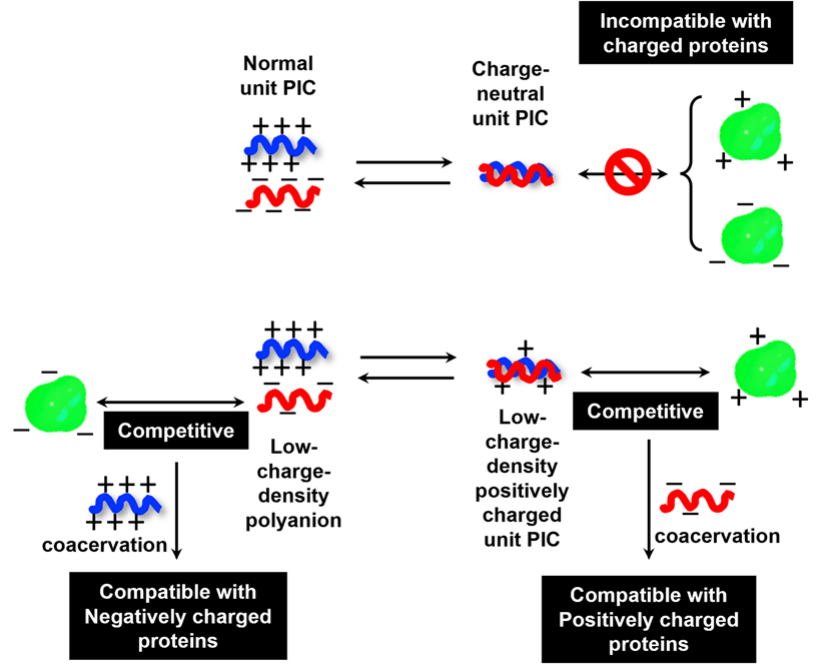
A proposed model for protein sequestration based on the unit PIC formation.

To investigate the selectivity regarding net charges of proteins, two isoforms of GFPs with net charges of −29 and +33 were prepared using the *E. coli* expression system (ESI Fig. S31†). Compared to native EGFP (net charges: −9), EGFPs (−29 and +33) were highly sequestered in both PEG-P(Asp-Pr-OH)-based and PEG-P(Asp-Bu)-based coacervates (ESI Fig. S32†). Therefore, there was no charge-sign selectivity, and only reduction of the linear charge density on the polypeptide governs the properties of complex coacervates as a scaffold to capture globular proteins as a client. To simplify the system for further discussion, postulating the formation of unit PIC is effective, which is a minimum-sized PIC, typically composed of a single aniomer and catiomer pair, as an elemental component of the entire assembly.^43–45^ In the present study, a PIC pair of a low charge density polyanion and a high charge density polycation yields a low-positively charged unit PIC (Fig. 3c and Scheme 1), which can easily interact with negatively charged proteins. Furthermore, low-positively-charged unit PICs can competitively interact with positively charged proteins for complexation with low-charge density polyanions. Therefore, the basic component of the scaffold, the low-charge-density positively-charged unit PICs, can act like natural low charge density IDPs. Because there is no selectivity in terms of net charges on proteins (ESI Fig. S29, S30, and Table S3†), this model can also explain the dependency of protein sequestration on the total number of charges rather than the net charges of proteins as an induced fit interaction of the coacervates with both positive and negative charges on the protein surface.

### Protein function preservation after sequestration

**Fig. 5:**
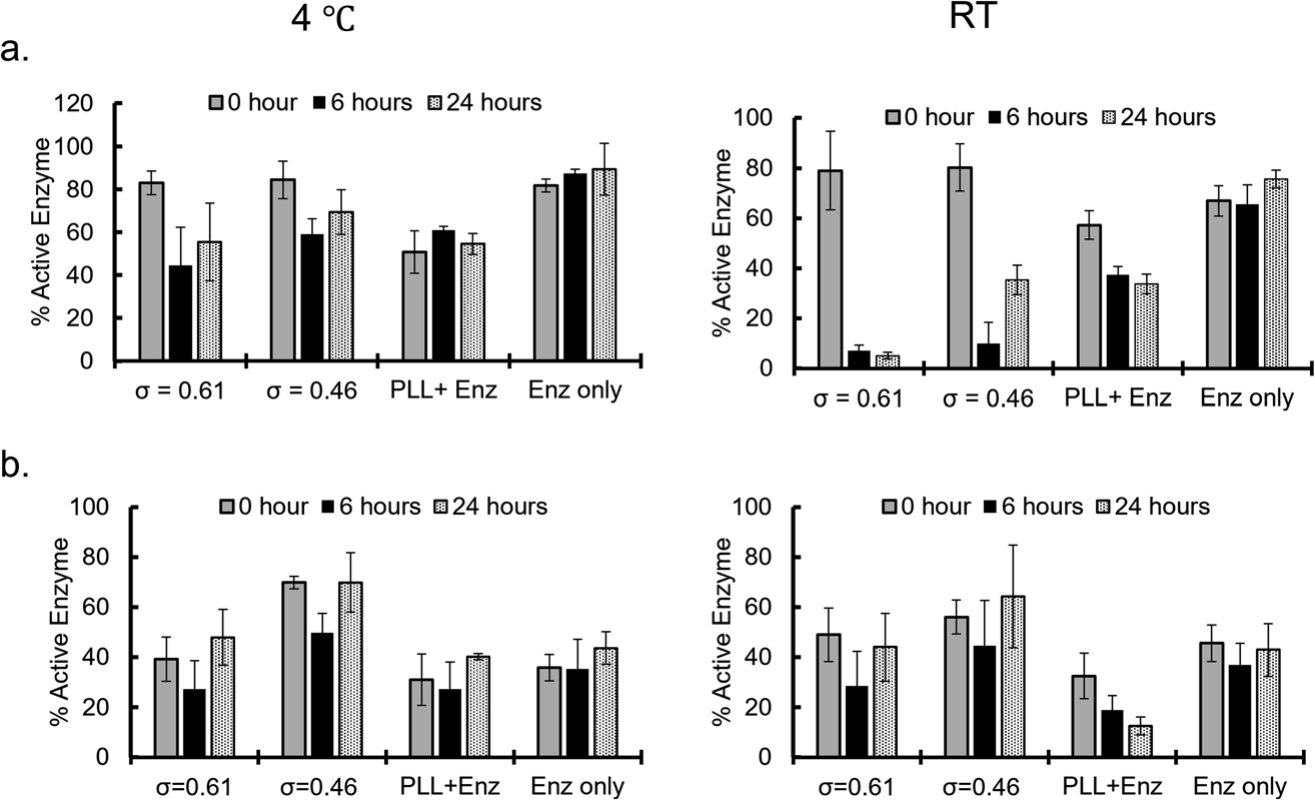
Active enzyme fractions (%; (a) β-galactosidase and (b) glucose oxidase) obtained for enzymes recovered from the coacervates after incubation at 4 °C (left) and room temperature (RT; right) for different time periods.

Enzymatic activities of the sequestered β-gal and GOx were measured at different incubation times after sequestration into PEG-P(Asp-Pr-OH)-based coacervates. Coacervates were incubated at 4 °C or RT (25–30 °C) and the activities were measured after dissolving coacervates to release the protein under high ionic strength buffer (2 M NaCl, 25 mM HEPES, pH 7.4). β-Gal activity was measured *via* the standard protocol of using *o*-nitrophenyl-β-galactopyranoside (ONPG) as a substrate. By comparing the initial velocity of the reaction to that of the standard samples of known enzyme concentration and encapsulation efficiency, the percentage of active enzyme in the coacervate was computed. The percentage of active enzyme decreased over the incubation period (Fig. 5a). This trend was more pronounced with relatively high incubation temperature; this is associated with the agglomeration of β-gal within the coacervate with prolonged incubation time and raised temperature, which can cause deactivation of enzymes. The enzymatic activity of GOx was evaluated using two step reactions with peroxidase as the secondary enzyme, and d-glucose and *O*-dianisidine as the substrates. The GOx activity appears to be well preserved even at relatively high incubation temperature as the percentage of active enzyme within the coacervate remains the same or exceeds that of the enzyme only sample (Fig. 5b and S33†). This preservation of enzyme activity would be consistent with the fact that GOx-loaded coacervates showed reduced agglomeration within the coacervate. Notably, the reduced percentage of active enzyme for the control group can be explained by the relatively high ionic-strength buffer treatment, which caused the deactivation of GOx. Therefore, coacervate might also contribute to the protection of enzymes from the harsh treatment.

### Dynamic behaviour of microenvironments in protein-sequestered coacervates

**Fig. 6:**
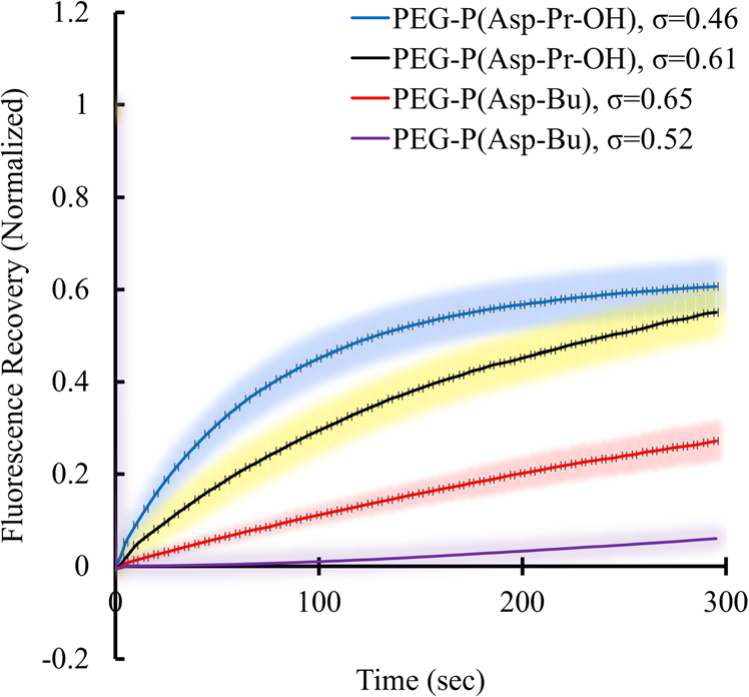
FRAP results of Cy3-BSA for different *σ*-reduced coacervates showing dependency on side-chain functionalities of polypeptides. Colour smear region indicate ±SD.

Condensed polymer solutions including biomolecular condensate are inherently viscoelastic, and the analysis of such property offers insight into the function of biomolecular condensates in *in vivo* situations. Therefore, the liquid-like or gel-like properties of coacervates should be correlated with the dynamic nature of molecular assemblies *e.g.*, mobility of constituent molecules within coacervates. First, the mobilities of polymers and proteins within the coacervate droplets regarding the varied *σ* of polyions were evaluated. These elucidate the effect of the sequestered protein on the mobility of constituent polymers of coacervates. The fluorescence recovery after photobleaching (FRAP) measurements were performed to probe protein and polycation mobility within coacervates using Cy3-labelled protein or polymers. Surprisingly, the mobility of protein in BSA-loaded coacervates differed from that in PEG-P(Asp-Pr-OH)-based and PEG-P(Asp-Bu)-based coacervates. The mobility of BSA was enhanced as the *σ* for PEG-P(Asp-Pr-OH)-based coacervate decreased (Fig. 6). An opposite trend was observed for PEG-P(Asp-Bu)-based coacervates, where slower mobility of BSA was observed for coacervates with lower *σ*. This difference can be explained by the lower relative permittivity of the PEG-P(Asp-Bu)-based coacervate, which retards the motion of rather hydrophilic BSA in the coacervate. Measurement of the mobility of one of the scaffold polymers, Cy3-PLL, in the absence of protein, showed only a slight difference in mobility for both PEG-P(Asp-Pr-OH)-based and PEG-P(Asp-Bu)-based coacervates with different *σ* (ESI Fig. S34a†). Conversely, in the presence of BSA, the mobility of Cy3-PLL was found to be significantly low for PEG-P(Asp-Bu) with relatively low *σ* (*σ* = 0.52; ESI Fig. S34b†). These results showed that the dynamic nature of coacervates could be altered after protein sequestration. Particularly for PEG-P(Asp-Bu) (*σ* = 0.52), a phase transition to gelation possibly occurred, where the secondary interaction of proteins with chemical moieties of scaffold polymers played a critical role. At relatively high ionic strength condition (150 mM NaCl), the mobility of BSA was recovered to a certain extent, indicating the key role played by the balance between hydrophobic and hydrophilic microenvironments (ESI Fig. S35†). Notably, in the case of β-gal, there was no recovery of the protein fluorescence in the photobleached region for both PEG-P(Asp-Pr-OH)-based and PEG-P(Asp-Bu)-based coacervates, which is consistent with an agglomeration of β-gal inside the coacervate (ESI Fig. S36†). This kind of change in the viscoelastic nature of droplets after sequestration of protein might be relevant in biological condensate where undesired partitioning of protein can alter its nature.

Therefore, there are two different aspects of side-chain modification on the polypeptide in terms of molecular level interaction. First, relatively low *σ* increases in transient non-neutralized charges along the polyion chains; in other words, dynamic and frustrated hotspots appear in the PIC domain (Fig. 3b and c). This dynamic charge imbalance provides the source of long-range interaction, which is responsible for the accommodation of charged proteins as mentioned above. The second aspect is short-range interaction of the side-chain of polyions with the protein, which can occur after the initial electrostatic-interaction and stabilize the entire complex. Therefore, the dynamic microenvironments within coacervates composed of charge-density-unmatched PICs dictates its protein sequestration behaviour.

## Concluding remarks

In summary, by reducing the charge density on a polypeptide, spontaneous sequestration of proteins into complex coacervates was achieved with very high encapsulation efficiency. This simple strategy can be used in wider fields and show potential utility for future applications. Remarkably, in this sequestration behaviour, proteins do not affect the polymer composition of the coacervate, which is similar to the scaffold-client interactions found in biomolecular condensates. Given that natural IDPs have low charge density, the charge-reduced polypeptide-based complex coacervate could provide a remarkable synthetic model for elucidating the physical process happening in such a biological system. Furthermore, considering charge neutrality in the coacervate phase, the coacervate can act as a solvent, where dynamic electrostatic interactions were responsible for sequestering proteins. This perspective may be beneficial in the examination of the nature of the biomolecular condensate, particularly if their properties can be traced back to the designable parameters of the scaffolds *e.g.*, the linear charge density of the scaffold or side-chain functionalities.

From the viewpoint of physical properties of coacervates, reduced charge density *via* side-chain modification with neutral group results reduced the numbers of ion pairs, which is a similar effect found *via* the increment of ionic strength. Moreover, secondary short-range interaction provided by the side-chain functionality can alter the nature of the coacervate microenvironment, which affects protein states, particularly further phase separation observed as agglomeration, and the difference in dynamic behaviour of component molecules within coacervates. Furthermore, after protein partitioning, the dynamic nature of polymers was changed in the coacervates underlying the influence of client recruitment on the physical nature of biomolecular condensate. From an engineering perspective, the fine-tuning of interactions between proteins and scaffold might present new frontiers in the design of artificial membrane-less organelles. Our previous studies have shown that polyion complexes can be finely tuned by introducing different functionalities in the polyions to make them salt- or temperature-resistant.^46–50^ Coupling such approaches with the present findings is promising to expand the potential utility of the synthetic-coacervate-based system for more robust applications. Although further investigations on structure–property relationship and physical properties and considerations on the engagement of various client molecules are required for rational design, our present findings offer key insights into the future progress of material and life sciences.

## Experimental

Details about the materials, polymer synthesis and modifications, protein expressions and purifications are in the ESI.† Also details on general methods like DLS, TEM, Microscopic Imaging are in electronic ESI.†

### Preparation of polymer and protein solutions

All the polymer solutions were prepared with HEPES buffer. Stock solution of 20 mg mL^−1^ polymer solution were prepared in 50 mM HEPES at pH 7.4 buffer and stored at −30 °C freezer. Prior to use, the working solution of 5 mg mL^−1^ at 25 mM HEPES was made and filtered using 0.22 μm PVDF syringe filter (Acrodisc, Pall Corporation, NY, USA). Depending on the experimental condition, salt concentration was varied by using 2 M NaCl solution prepared using 25 mM HEPES solution. All the protein solutions were also stocked at −80 °C in aliquots and working concentration of 0.5 mg mL^−1^ were made in 25 mM HEPES buffer, followed by filtration using 0.22 μm PVDF syringe filter (Acrodisc, Pall Corporation, NY, USA).

### Coacervate preparation and microscopic observation

To prepare coacervates in the absence of protein, 25 μL of polyanion solution were mixed with a proper volume of PLL with the cation to anion ratio (C : A) 1 : 1 *via* gentle pipetting. The final volume was adjusted to 120 μL by adding a proper buffer. Salt concentration was adjusted using a 2 M NaCl buffer before mixing polymer solutions. The mixture was equilibrated at 4 °C, at least, for 3 h followed by centrifugation at 1000 G, 10 min (KUBOTA 5922, Kubota Corporation, Osaka, Japan). The supernatant (∼100 μL) was carefully aspirated and used for DLS measurement (Malvern ZETASIZER PRO, Malvern Panalytical Ltd, Malvern, UK) and TEM (JEM-2010, JEOL, Ltd, Japan) observation. The formation of coacervate was verified by visual observation of distinct macro-phase separation in the tube and microscopic observation of the distinct droplet formation by brightfield imaging using a BZ-X810 microscope (Keyence Corporation, Osaka, Japan) with 40× objective lens (PlanApoλ NA 0.95, Nikon, Tokyo, Japan). For microscope observation, first, coverslip (22 × 40 mm, 0.17 mm thickness) was attached to the pre-cleaned standard glass-slide (S2215, Matsunami) using two-sided tape to have little gap in between glass-slide and coverslip. Then, approximately 20 μL of dispersed coacervate solution was loaded into the gap and observed under the microscope.

### Critical salt concentration determination

Critical salt concentration for dissociation (Cs_dis_) was defined as the concentration of salt at which the coacervate disappeared and the solution became transparent. Cs_dis_ was measured as the increase in percent transmission as the function of increasing NaCl concentration. First, homoPLL-HBr and different polyanions were mixed at C : A 1 : 1 (total volume 300 μL). Then, 50 μL of coacervate dispersion was mixed with 50 μL of buffer containing a series of concentrations of NaCl (0–800 mM). For each sample, transmittance (%*T*) was measured at 600 nm using JASCO V-670 UV-Vis Spectrophotometer (Jasco International Co. Ltd, Tokyo, Japan) and plotted against increasing NaCl concentration. Three independent readings were made. The plotted curve was fitted with a higher order polynomial function whose first derivative maxima after normalization was used to obtain Cs_dis_.

### Protein sequestration into coacervate droplets

To encapsulate proteins during the coacervate formation process, 12.5 μL of labelled protein and 25 μL of polyanion solution were mixed in 25 mM HEPES buffer. An appropriate volume of PLL solution was added to the mixture at C : A 1 : 1 *via* gentle pipetting approximately 10 times. The final volume of mixture was maintained at 120 μL in all samples by adding additional buffer prior to adding PLL solution. The resulting mixture was left to be equilibrated at 4 °C for 3 h followed by centrifugation at 1000 G at 4 °C for 10 min. The supernatant was used to measure fluorescence intensity using an Infinite Pro200, TECAN M PLEX plate reader (Tecan Japan Co. Ltd, Osaka, Japan). After obtaining the supernatant concentration value from a standard curve prepared from a series of known concentration samples, encapsulation efficiency was calculated as follows (eqn (i)):i
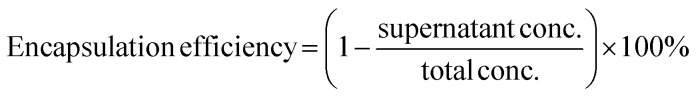


The collected coacervate sample was observed using a TCS SP8 STED CLSM (Leica Microsystems, Danaher Co., USA) or a fluorescence microscope (BZ-X810 KEYENCE) to see the sequestered protein. For microscope observation, first, a coverslip (22 × 40 mm, 0.17 mm thickness) was attached to the precleaned standard glass-slide (S2215, Matsunami) using two-sided tape to have little gap in between glass-slide and coverslip. Then, approximately 10 μL of dispersed coacervate solution containing labelled protein was loaded into the gap and observed under the microscope.

### Quantification of polymers consumed for coacervation and determination of charge ratios

Coacervates were prepared using 2% (v/v) labelled polyions, FITC-labelled PLL and rhodamine-labelled polyanions as mentioned above with protein (β-gal) and without protein (buffer). For polyanions with *σ* = 1.0, final NaCl concentration of 400 mM, for *σ* > 0.7, 150 mM and for *σ* < 0.7, 0 mM were used for effective micrometer-sized droplet formation. After centrifugal separation, fluorescence intensity of the supernatant was measured using an Infinite Pro200, TECAN M PLEX plate reader (Tecan Japan Co. Ltd, Osaka, Japan) and concentration of polymers in the supernatant was determined by using a standard curve prepared from a series of known concentration samples. The amount of polyions in the coacervates was estimated as follows (eqn (ii)), assuming equal total volume in all cases:ii
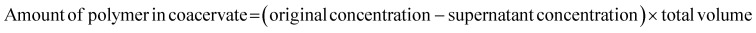
iii
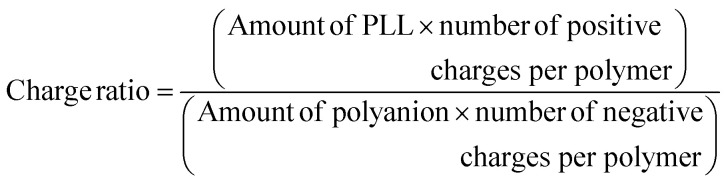


Charge ratio was computed based on the estimated polymer (in moles) present in the coacervate (eqn (iii)).

### Reversibility of protein encapsulation

Rho-β-gal loaded coacervate sample was prepared for PEG-P(Asp-Pr-OH), *σ* = 0.46 as described as above. After equilibration for 3 h, 4 °C, 10 μL of sample was taken for microscope observation and remaining dispersed sample was transferred to the microdialzer tube (DiaEasyTM Dialyzer K1021, MWCO 12 000–14 000, Biovision, Inc., CA, USA). Then the sample was dialyzed under 150 mM NaCl, 25 mM HEPES buffer (pH 7.4) overnight and observed under microscope, followed by overnight dialysis with 0 mM NaCl, 25 mM HEPES buffer (pH 7.4), and microscopic observation.

### Hexane-1,6-diol assay

Rho-β-gal loaded coacervate sample was prepared for PEG-P(Asp-Pr-OH), *σ* = 0.46 with FITC labelled PLL (2% v/v) and FITC-BSA loaded coacervate for PEG-P(Asp-Bu), *σ* = 0.52 as described above. After equilibration for 3 h at room temperature, and centrifugation at 1000 G, 10 min, the supernatant was exchanged with the buffer containing varying percentages of hexane-1,6-diol (4%, 20% (w/v)) and mixed *via* gentle pipetting approximately 10 times. 20 μL of the dispersed sample was observed under the fluorescence microscope.

### Size-exclusion chromatography of the coacervate to profile released protein

First coacervate was prepared at C : A 1 : 1 with and without protein (same total volume) for charge reduced coacervate, and centrifuge at 300 G, 10 min after 3 h equilibration. The dilute phase was changed with 500 mM NaCl buffer, 10 mM PB to dissolve the coacervate phase. The SEC profiles were obtained by using JASCO HPLC system, UV-4075 detector equipped with SuperdexTM 200 10/300 GL column (Cytiva, Massachusetts, USA) with 10 mM PB, 500 mM NaCl pH 7.4 as eluent at flowrate of 0.5 mL min^−1^ for 60 min. The protein detection was made at UV 280 nm wavelength and normalized (by maxima) to compare the chromatograms.

### Enzyme activity measurement

For enzymatic activity, after protein sequestration, the 100 μL of supernatant was exchanged with fresh buffer. The supernatant was used to determine the encapsulation efficiency for each individual tube as described above. The sample was incubated at 4 °C or room temperature (RT) for different time periods. After each period of time, 10 μL of coacervate dispersion was pipetted out after adequate mixing and added to 90 μL of 2 M NaCl, 25 mM HEPES solution. After dissolving the coacervate, the activity of the enzyme was measured following the standard protocol. β-Gal activity was measured using ONPG as substrate.^51^ In brief, 160 μL of Z buffer with pH 7.2 (with 0.04 M β-mercaptoethanol) was mixed with 20 μL of 4 mg mL^−1^ ONPG solution and 20 μL of sample/standard enzyme in 96 well plate. Then, the absorbance measurement at 420 nm was taken for 30 min at 37 °C using an Infinite Pro200, TECAN M PLEX plate reader (Tecan Japan Co. Ltd, Osaka, Japan). The initial velocity of the reaction was computed as slope from the linear fit of absorbance *vs.* time data and compared with the standard known enzyme concentration data to determine the concentration of active enzyme in the sample. This value was converted to the percentage of active enzyme from the total encapsulated protein amount. For the enzymatic activity of GOx, following the manufacturer's protocol, first, 19.2 mL of 0.21 mM *O*-dianisidine solution (in 50 mM acetate buffer, pH 5.1) was mixed with 4 mL of 10% (w/v) d-glucose solution (in UPW) to make reaction cocktail. Then, 145 μL of freshly prepared reaction cocktail (prewarmed to 35 °C) was mixed with 5 μL of peroxidase enzyme solution (POD type II) (60 units per mL) and 5 μL of standard GOx enzyme solution or sample in 96 well plate. Absorbance read at 500 nm was taken over 15 min at 35 °C using an Infinite Pro200, TECAN M PLEX plate reader (Tecan Japan Co. Ltd, Osaka, Japan). Using a similar procedure as β-gal, percentage of active enzyme was computed from initial reaction velocity. All readings were performed in triplicate for each of the three independent experiments.

### Fluorescence recovery after photobleaching measurement

Cy3-labelled PLL (2% v/v) or Cy3-labelled proteins were used for fluorescence recovery after photobleaching (FRAP) measurement. The coacervate sample was prepared as mentioned before. After centrifugation, the supernatant was exchanged with fresh buffer and the coacervate droplets were observed under CLSM (Leica TCS SP8 STED). For FRAP, fly in mode was used where droplets more than 20 μm in diameter were selected and a small region of interest was bleached with 100% Ar laser light (Ar-515 nm) for 30 frames (1.54 s per frame) and then 65 frames with 5 s per frame were taken subsequently as time-lapsed images with PMT detector. The FRAP data were analyzed using ImageJ (Stowers Plugin).

### Statistical analysis

Student's *t*-test was used to determine the statistical difference between experimental groups. *P*-Value < 0.05 was considered statistically significant.

## Data availability

All relevant data are within the manuscript and its ESI files.†

## Author contributions

BKC carried out all the experiments, analyzed the data and wrote the manuscript (investigation, methodology, formal analysis, validation, writing-original draft and writing-review and editing). TN, TM and YK co-supervised the research and contributed to the design of the experiments and manuscript preparation. AK designed the experiments, supervised the research, analyzed the data and wrote the manuscript (conceptualization, project administration, supervision, methodology, formal analysis, writing-original draft and writing-review and editing).

## Conflicts of interest

The authors declare no competing financial interest.

## Supplementary Material

SC-014-D3SC00993A-s001

SC-014-D3SC00993A-s002

SC-014-D3SC00993A-s003

SC-014-D3SC00993A-s004
