# Supplementary material for: Dynamic frustrated charge hotspots created by charge density modulation sequester globular proteins into complex coacervates
Source: Chem Sci. 2023 May 19;14(24):6608–20. doi: 10.1039/d3sc00993a (PMC10283495; doi:10.1039/d3sc00993a)
Supplement: SC-014-D3SC00993A-s004 [file SC-014-D3SC00993A-s004.pdf]

Electronic Supplementary Information

Dynamic frustrated charge hotspots created by charge density modulation sequester globular proteins into complex coacervates

Biplab K C[b], Teruki Nii[a], Takeshi Mori[ac], Yoshiki Katayama[acdef] and *Akihiro Kishimura[acdg]

a. Department of Applied Chemistry, Faculty of Engineering, Kyushu University, 744 Moto-oka, Nishi-ku, Fukuoka 819-0395, Japan
b. Graduate school of Systems Life Sciences, Kyushu University, 744 Moto-oka, Nishi-ku, Fukuoka 819-0395, Japan.
c. Centre for Future Chemistry, Kyushu University, 744 Moto-oka, Nishi-ku, Fukuoka, 819-0395, Japan
d. Centre for Molecular Systems, Kyushu University, 744 Moto-oka, Nishi-ku, Fukuoka, 819-0395, Japan
e. Centre for Advanced Medical Open Innovation, Kyushu University, 3-1-1 Maidashi, Higashi-ku, Fukuoka, 812-8582, Japan
f. Department of Biomedical Engineering, Department of Biomedical Engineering, Chung Yuan Christian University, 200 Chung Pei Rd., Chung Li, Taiwan, 32023 ROC
g. RIKEN Centre for Emergent Matter Science, 2-1 Hirosawa, Wako, Saitama 351-0198, Japan.

Please do not adjust margins

Table of Contents:

1. Materials: 6

2. General Methods: 6

^{1}H Nuclear Magnetic Resonance spectroscopy 6

Size Exclusion Chromatography 6

Dynamic Light Scattering 7

Transmission electron microscopy 7

Fluorescence Imaging 7

Refractive Index Measurement 7

3. Polypaptide Synthesis and Characterization 7

Scheme S1. Synthetic scheme for hydrolysis of PEG-PBLA to get PEG-PAsp 9

Fig. S1. ^{1}H NMR spectrum of PEG-PBLA in DMSO-d_6 at 25 °C. 9

Fig. S2. ^{1}H NMR of PEG-PAsp σ=1.0 (Scans=64) in D_2O, 400 MHz, 80 °C. 10

Table S1. Reaction condition for partial aminolysis for PEG-P(Asp-*Pr-OH*) 11

Scheme S2. Synthetic scheme for partial aminolysis of PEG-PBLA to get PEG-P(Asp-*Pr-OH*) 11

Fig. S3. ^{1}H NMR of PEG-P(Asp-*Pr-OH*), σ= 0.84 (Scans=64) in D_2O, 400 MHz, 80 °C, TMSP as internal reference. 11

Fig. S4. ^{1}H NMR of PEG-P(Asp-*Pr-OH*), σ = 0.74 (Scans = 64) in D_2O, 400 MHz, 80 °C, TMSP as internal reference. 12

Fig. S5. ^{1}H NMR of PEG-P(Asp-*Pr-OH*), σ = 0.69 (Scans = 64) in D_2O, 400 MHz, 80 °C, TMSP as internal reference. 12

Fig. S7. ^{1}H NMR of PEG-P(Asp-*Pr-OH*), σ= 0.46 (Scans=64) in D_2O, 400 MHz, 80 °C, TMSP as internal reference. 13

Fig. S6. ^{1}H NMR of PEG-P(Asp-*Pr-OH*), σ= 0.61 (Scans=64) in D_2O, 400 MHz, 80 °C, TMSP as internal reference 13

Table S2. Reaction condition for partial aminolysis for PEG-P(Asp-Bu) 14

Scheme S3. Synthetic scheme for partial aminolysis of PEG-PBLA to get PEG-P(Asp-*Bu*) 14

Fig. S8. ^{1}H NMR of PEG-P(Asp-*Bu*), σ= 0.87 (Scans=64) in D_2O, 400 MHz, 80 °C, TMSP as an internal reference. 15

Fig. S9. ^{1}H NMR of PEG-P(Asp-*Bu*), σ= 0.70 (Scans=64) in D_2O, 400 MHz, 80 °C. 15

Fig. S10. ^{1}H NMR of PEG-P(Asp-*Bu*), σ= 0.65 (Scans=64) in D_2O, 400 MHz, 80 °C, TMSP as an internal reference. 16

Fig. S11. ^{1}H NMR of PEG-P(Asp-*Bu*), σ= 0.52 (Scans=64) in D_2O, 400 MHz, 80 °C, TMSP as an internal reference. 16

Fig. S12. Size-exclusion chromatograms of hydroxyl modified polyions a. PEG-PAsp, b. PEG-P(Asp-*Pr-OH*), σ = 0.84, c. PEG-P(Asp-*Pr-OH*), σ = 0.74, d. PEG-P(Asp-*Pr-OH),* σ = 0.70, e. PEG-P(Asp-*Pr-OH*), σ = 0.61, and f. PEG-P(Asp-*Pr-OH*), σ = 0.46 17

Fig. S13. Size-exclusion chromatograms of butyl modified polyions. a. PEG-P(Asp-*Bu*), σ=0.87, b. PEG-P(Asp-*Bu*), σ=0.70, c. PEG-P(Asp-*Bu*), σ=0.65, d. PEG-P(Asp-*Bu*), σ=0.52. 17

This journal is © The Royal Society of Chemistry 20xx

Please do not adjust margins

Please do not adjust margins

Fig. S14. a. SEC profile of PEG-P(Asp-*Pr-OH*), σ=0.46. Three fractions were collected for further characterization. b. SEC profiles of each fraction. c. ^{1}H NMR spectra of each fraction (top) indicating similar degree of modification (Table in the bottom). 18

Fluorescence labelling of polymers 19

4. Protein Expression and Purification: 19

Preparation of GFPs 19

Protein Expression 19

Protein Isolation and Purification 19

Fluorescence labelling of proteins 20

5. Supporting Results: 21

Fig. S15. a. Mean count rate in supernatant determined from dynamic light scattering for different σ of propanol and butyl modified samples at 0 mM NaCl showing nano-assemblies formed for σ > 0.7. b. Transmission electron micrographs of the vesicle formed at low ionic strength (0 mM NaCl) for σ > 0.7. 21

Fig. S16. a. Mean count rate of the nano-assemblies determined by DLS in the supernatant of the polyion complex prepared at different NaCl concentration for PEG-P(Asp-*Pr-OH*) based complex coacervate. b. Tabulated values of optimal salt concentration for coacervation (macro-phase separation) determined by lack of formation of nano-assemblies from DLS measurement. 22

Fig. S17. a. Schematic diagram of side-chain modification by non-charged moieties and its impact on the number of ion-pairs in a polyion complex. b. Schematic illustration of the evaluation of critical salt concentrations by turbidity measurement using %Transmittance at 600 nm. c. Salt-concentration-dependency of %Transmittance of charge-reduced-polyion-based coacervate from PEG-P(Asp-*Pr-OH*) (left) and PEG-P(Asp-*Bu*) (right). d. Polynomial fittings and 1st order derivative curves obtained from %Transmittance curves to determine critical salt concentrations for various σ. 23

Fig. S18. Refractive indices (n) of coacervate droplets for different sizes ranging from 1 to 10 µm obtained using Xsight. [Coacervates prepared from PEG-P(Asp-*Bu*) (σ =0.52, red) and PEG-P(Asp-*Pr-OH*) (σ=0.46, black) at 25mM HEPES (pH 7.4, 0 mM NaCl).] 24

Fig. S19. Fluorescence image of coacervate droplets [σ = 0.61, PEG-P(Asp-*Pr-OH*)] prepared in the presence of Cy5-BSA (red) and fluorescein-β-gal (green). (Scale bars: 50 µm) 25

Fig. S20. Fluorescence and merged images of coacervate droplets prepared in the presence of FITC-BSA (green) and 50mM NaCl. 25

Fig. S21. Merged images of fluorescent and bright-field images of coacervate droplets prepared from PEG-P(Asp-*Pr-OH*) with high σ in the presence of rhodamine-labelled β-gal (red) and fluorescein-labelled BSA (green). (Scale bars = 20 µm) 26

Fig. S22. Remaining amounts (%) of PLL and modified PEG-PAsp with different σ in the coacervate. (a, b) For PEG-P(Asp-*Pr-OH*)-based coacervates, and (c, d) for PEG-P(Asp-*Bu*)-based coacervates. 27

Fig. S23. a. Concentration-dependent change in % encapsulation efficiency of BSA for the coacervate prepared from PEG-P(Asp-*Pr-OH*) with σ = 0.46. b. Polyion-to-protein ratio in the coacervate at different BSA concentrations. 27

This journal is © The Royal Society of Chemistry 20xx

Please do not adjust margins

Please do not adjust margins

Fig. S24. a. Fluorescence images of the complex coacervates prepared from FITC-PLL (green), PEG-P(Asp-*Pr-OH*) with σ = 0.7, and rhodamine labelled β-gal (red) at different NaCl concentrations. b. Effect of NaCl concentrations after protein (rhodamine labelled β-gal, red) loading into coacervates prepared from PEG-P(Asp-*Bu*) or PEG-P(Asp-*Pr-OH*) with σ = 0.7. 28

Fig. S25. Size exclusion chromatograms of the various coacervates from polyanions with different σ after the treatment with high-ionic-strength buffer (500 mM NaCl, 10 mM PB). The chromatograms for the coacervates with or without loading proteins were compared with that of the pure protein. 29

Fig. S26. Fluorescence images of coacervate droplets encapsulating proteins after the hexane-1,6-diol treatment with different conditions. a. Coacervates were prepared from PEG-P(Asp-*Pr-OH*) with σ = 0.61, where Rhodamine-β-gal (rho-β-gal, red) was loaded, were treated with 4 and 20 wt% hexane-1,6-diol. b. Coacervates were prepared from PEG-P(Asp-*Bu*) with σ=0.52 and 0.65, where FITC-BSA (green) was loaded, were treated with 0 and 20 wt% hexane-1,6-diol. Scale bars: 50 µm 30

Fig. S27. A typical image of protein agglomeration within the PEG-P(Asp-*Pr-OH*), σ=0.46 and PEG-P(Asp-*Bu*)-based coacervate, σ=0.52 encapsulating Cy3-β-gal (red) and Cy3-BSA (green, false colour for clarity) incubated overnight at 4 °C. White arrows showed the agglomerated granules within the liquid coacervate. 30

Figure S28. Fluorescence images of PEG-P(Asp-*Pr-OH*)-based coacervates encapsulating rho-β-gal (red) just after preparation at 4 °C (left), after 6-h equilibration at 4 °C (middle), and 6-h equilibration at room temperature (right). The increase of bright spots in the droplets indicates agglomeration of rho-β-gal. 31

Table S3: Different proteins used for encapsulation study and their information on total number of charges and molecular weights (MW). 31

Table S4: Comparison of charge/mass ratio (e/kDa) for different proteins and polyions used in this study. 31

Fig. S29. Fluorescence images of charge-reduced-polymer-based complex coacervate droplets prepared in the presence of proteins (EGFP (green), Lysozyme (rhodamine labelled, red) and IgG (rhodamine labelled, red)). 32

Fig. S30. Fluorescence images prepared from charge-reduced-polymer-based complex coacervate droplets in the presence of proteins (Gox (FITC labelled, green), HRP (rhodamine labelled, red) and DaO (cy5 labelled, cyan)) 33

Fig. S31. Preparation of GFP isoforms. a. Schematic flow of the protein expression in E. coli. b. Information on GFP isoforms. Molecular weight (MW), theoretical p*I* (Th. p*I*), and net charges. c. His-Tag column elution profiles for EGFP with the buffer B (20 mM Tris HCl, 0.5 M NaCl, 0.5 M imidazole, pH 7.4) d. Coomassie-blue stained SDS-PAGE for EGFP and GFP (+33) e. Coomassie-blue stained SD-PAGE of different fraction of GFP (-29) after size-exclusion chromatography purification (only fractions 16-21 were collected.) f. Native PAGE of the different isoforms of GFPs. GFP (-29) moved further compared to EGFP. No band in GFP (+33) was confirmed due to opposite direction of mobility in the gel g. Emission spectra of GFP isoforms with excitation at 488 nm. 34

Fig. S32. Encapsulation efficiency (%) of charge-reduced-polymer-based coacervates for three isoforms of GFPs. 35

Fig. S33. Active enzyme fraction (%) recovered from the PEG-P(Asp-Bu)-based coacervates when stored at a. 4 °C and b. room temperature for glucose oxidase. 36

Fig. S34. FRAP results for Cy3-labelled PLL using various charge-density-reduced coacervates. a. Without protein, and b. with protein (BSA). PLL mobility reduced significantly at higher butyl modification in the presence of protein. Smear colour region indicate ±S.D. 37

 This journal is © The Royal Society of Chemistry 20xx

Please do not adjust margins

Please do not adjust margins

Fig. S35. FRAP results of Cy3-BSA loaded PEG-P(Asp-*Bu*)-based coacervate (σ=0.52) prepared at different NaCl concentrations. Mobility of Cy3-BSA increased at 150 mM NaCl compared to that at 0 mM NaCl. Smear colour region indicate ±S.D 37

Fig. S36. FRAP results of Cy3-labelled β-gal loaded in coacervates prepared from PEG-P(Asp-Pr-OH), σ=0.46, and PEG-P(Asp-Bu), σ=0.52. 38

Movie S1 (.avi). Time-lapsed images of sequestration of rho-β-gal (red) into the coacervate prepared from PEG-P(Asp-*Pr-OH*), σ=0.46. 38

Movie S2(.avi). Time-lapsed images of sequestration of FITC-BSA (green) into the coacervate prepared from PEG-P(Asp-*Pr-OH*), σ=0.46. 38

Movie S3 (.mp4). Sequestration of rho-β-gal (red) into the FITC-BSA (green) pre-loaded coacervate prepared from PEG-P(Asp-*Pr-OH*), σ=0.46. 38

6. References: 38

This journal is © The Royal Society of Chemistry 20xx

Please do not adjust margins

1. Materials:

α-Methoxy-ω-propylamine poly (ethylene glycol) (MeO-PEG-NH_2; Mw ~ 2,000) was purchased from Yuka Sangyo Co., Ltd. (Tokyo, Japan) and purified via ion exchange chromatography using CM Sephadex C-50 (Sigma; St. Louis, MO, USA). β-Benzyl-L-aspartate N-carboxy-anhydride (BLA-NCA) was purchased from Chuo Kaseihin Co., Inc. (Tokyo, Japan). Poly-L-lysine hydrobromide (average degree of polymerization (DP) = 108) was purchased from Sigma Aldrich (St. Louis, MO, USA), and dissolved in UPW and freeze-dried prior to use. n-Butylamine (Special Grade) were purchased from Wako Pure Chemical Co., Ltd. (Osaka, Japan) and was refluxed (3 h) and distilled over CaH2 at 78 °C under N_2 atmosphere. 3-Amino-1-propanol (>99%) was purchased from Tokyo Chemical Industry Co., Ltd. (TCI) (Tokyo, Japan) and dehydrated via azeotropic evaporation with 1,4 dioxane in vacuo. *N,N*-dimethylformamide (DMF) (super dehydrated), dichloromethane (DCM) (super dehydrated), hexane and ethyl acetate were purchased from Kanto Chemical Co. Inc., (Tokyo, Japan). Di-isopropyl ethyl amine (DIPEA) was purchased from Sigma Aldrich (St. Louis, MO, USA) and used as such. Chloroform ($CHCl_3$) (super dehydrated) and benzene (super dehydrated) were purchased from Wako Pure Chemical Co., Ltd. (Osaka, Japan). Deuterium oxide (D_2O) was purchased from Sigma Aldrich (St. Louis, MO, USA). Deuterated dimethyl sulfoxide (DMSO-d_6) was purchased from MagniSolvTM, EMD Millipore corporation (MA, USA). Pre-wetted Spectra/Por® regenerated cellulose (RC) membrane (molecular weight cut-off (MWCO) 3,500) were purchased from Spectrum Laboratories, Inc., (Rancho Dominguez, CA, USA). PTFE membrane filter (0.2 µm pore size) was purchased from Advantec Inc., (Tokyo, Japan). β-D-Galactosidase (β-gal) (EC 3.2.1.23) (Reagent Grade) was purchased from Wako Pure Chemical Co., Ltd. (Osaka, Japan) and stored at 4 °C prior to use. Lyophilized Bovine Serum Albumin (BSA) and Horse Radish Peroxidase (HRP) were purchased from Sigma Aldrich (St. Louis, MO, USA) and stored at 4 °C prior to use. Lyophilized powder of Glucose Oxidase (GOx) was purchased from Nacalai Tesque Inc., (Kyoto, Japan) and stored at -30 °C prior to use. Diamine Oxidase (DaO) from porcine kidney (D7876-1G) was purchased from Sigma Aldrich (St. Louis, MO, USA) and stored at -30 °C prior to use. IgG (Ofatumumab) was purchased as Arzerra (I.V infusion 100 mg) from Novartis Pharma K.K. (Tokyo, Japan) and stored at 4 °C prior to use. N-hydroxy-succimide (NHS)-functionalized rhodamine dye (NHS Rhodamine) was purchased from Thermo Scientific, Germany. Cy3-NHS and Cy5-NHS were purchased from Funakoshi Co., Ltd (Tokyo, Japan). Fluorescein labelled isothiocyanate (FITC) was purchased from Invitrogen, Thermo Fischer Scientific Co., (CA, USA). PD-10 desalting columns (SephadexTM G-25) were purchased from (Cytiva, Global Lifescience Solutions, UK). O-dianisidine and D(+)-Glucose were purchased from Tokyo Chemical Industries (TCI), Tokyo. β-mercaptoethanol and O-nitrophenyl-β-D-galactopyranoside (ONPG) were purchased from Wako Pure Chemical Co., Ltd. (Osaka, Japan). Carbon coated Cu-grid (ELS-C10) was purchased from Okenshoji Co. Ltd. (Tokyo, Japan), and 70% Glutaraldehyde EM grade from Electron Microscopy Science (Hatfield, PA, USA).

Supercharged DNA sequences of Green Fluorescence Protein (GFPs) were obtained from the study performed by Nojima *et al.*[1] cDNA of GFP sequence were purchased from Integrated DNA Technologies, Inc. After purchase, DNA sequences were dissolved in TE buffer to make 10 ng/µL, heat treated (50 °C, 20 min) and stored at -30 °C. Primers were ordered from FASMAC Co. Ltd. (Kanagawa, Japan) in TE buffer at 100 µM concentration, diluted 10 times, and stored at -30 °C. Luria Bertani (LB) medium was purchased from Kanto Chemical Co. Inc. (Tokyo, Japan). Bacto Agar was purchased from Becton, Dickinson and Company (MD, USA). Buffers QG, P1, P2, N3, PE, and EB were purchased from QIAGEN (Hilden, Germany). FAPD column for DNA extraction as purchased from Chiyoda Science Co., Ltd. (Tokyo, Japan). Ampicillin Sodium was purchased from Fujifilm Wako Pure Chemical Co., Ltd. (Osaka, Japan) and stored at 4 °C. Agarose Kanto S was purchased from Kanto Chemical Co. Inc. (Tokyo, Japan). Isopropyl-beta-D-thiogalactopyranoside (IPTG) was purchased from MStechno Systems, Inc. (Osaka, Japan). NuPAGE LDS sample loading buffer (4×) was purchased from Invitrogen, Thermo Fischer Scientific Co., (CA, USA). PierceTM BCA protein assay kit was purchased from Thermo Scientific (Rockford, IL, USA).

2. General Methods:

^{1}H Nuclear Magnetic Resonance spectroscopy

^{1}H- Nuclear Magnetic Resonance (NMR) spectra of the polymer samples were recorded using a JNM-ECZ400 (400 MHz, JEOL, Ltd., Tokyo, Japan), where sample solutions were prepared at a concentration of 10 mg/mL using appropriate deuterated solvents. TMS or TMSP were used as an internal reference (0 ppm). Peak integrations were computed using Delta software (version 5.0.5.1, JEOL, Ltd.) and subsequently, number average degree of polymerization and number average molecular weight were calculated.

Size Exclusion Chromatography

The analytical Size Exclusion Chromatography (SEC) measurement of synthesized polymers were carried out using a high-performance liquid chromatography (HPLC) system (JASCO International Co., Ltd., Tokyo, Japan) with PU-4180 pump and UV-4075/ RI-4030 detectors (JASCO International Co., Ltd., Tokyo, Japan) equipped with a SuperdexTM 200 10/300 GL column (Cytiva, Uppsala, Sweden) where the 10 mg/mL sample were eluated using an aqueous phosphate buffer (PB solution) (10 mM PB, pH 7.4) containing 500 mM NaCl as the eluent at flowrate of 0.5 ml/min for 60 min. Polydispersity (Mw/Mn) (PD) was computed using the

 This journal is © The Royal Society of Chemistry 20xx

Please do not adjust margins

calibration curve from standard PEO of known molecular weights (ReadyCal-Kit PEO/PEG, PSS, Range (Da) Mp 232-1,015,000, Scharlab, Barcelona, Spain).

Preparative SEC of synthesized polymers and proteins were performed using a BioLogic DuoFlowTM Chromatography System (Bio-Rad Laboratories, Inc., CA, USA) equipped with an AVR7-3 sample injection valve, BioFracTM fraction collector, BioLogic QuadTecTM UV/Vis detector, F10 pumphead workstation and a HiLoad 16/600 Superdex 75 pg column (Cytiva, Uppsala, Sweden) at flow rate of 1 ml/min using 10 mM PB, 500 mM NaCl (pH 7.4) as an eluent. Then a 3 mL sample at a concentration of 10 mg/mL was injected for a single analysis and collected in pre-cleaned test-tubes.

Dynamic Light Scattering

The supernatant (~100 µL) of the prepared coacervate sample was carefully aspirated after centrifugation and used for dynamic light scattering (DLS) measurement. DLS was carried out at room temperature in ZEN2112 quartz cuvette on a Malvern ZETASIZER PRO (Malvern Panalytical Ltd., Malvern, UK) equipped with a 4 mW He-Ne 632.8 nm laser measured at 173° backscattering angle. The hydrodynamic diameters and polydispersity indices (PDIs) of the particles were calculated using the cumulant method using in-built software of the machine.

Transmission electron microscopy

Samples of nano-sized assembly detected by DLS were, first, cross-linked with 30 eq. of 25% glutaraldehyde to primary amines of PLL for 1 h at 4 °C in the dark, and then, purified using a PD-10 column with UPW as an eluent. An aliquot of 2 µL was placed on a 150-mesh copper grid coated with a thin film of formvar and reinforced with a carbon coating (ELS C-10, grid pitch 100 µm), and hydrophilized using an HDT-400 hydrophilic treatment device (JEOL, Ltd.) and left for 2 min followed by staining using 2% (wt/v) gadolinium acetate (containing 50% MeOH) for 2 min. Any excess solution was removed carefully using a piece of filter paper, and the grid was air-dried at room temperature. Then, the transmission electron microcopy (TEM) was performed using a JEM-2010 (LaB6 thermal cathode emission at 120 kV), and a micrograph was captured with a CCD camera cooled at -20 °C.

Fluorescence Imaging

Fluorescence microscopic observations of samples were performed using a BZ-X810 microscope (Keyence Corporation, Osaka, Japan) equipped with a light emitting diode (3.7 W), a metal halide lamp (80 W) with 40× Objective lens (PlanApoλ NA 0.95, Nikon, Tokyo, Japan) and dichroic mirror filters viz. OP-87764 BZX filter-TRITC (Excitation bandwidth 545/25 nm, Emission bandwidth 605/70 nm) and OP-87763 BZX filter-GFP (Excitation bandwidth 470/40 nm, Emission bandwidth 525/50 nm) for appropriate dyes.

Confocal laser scanning microscope (CLSM) observations were performed using a TCS SP8 STED microscope (Leica Microsystems, Danaher Co., USA) equipped with a white light laser (λex = 550 nm for rhodamine dye, 488 nm for GFPs and FITC, and 525 nm for Cy3 using a 40× objective lens (HC PL APO CS2 40× 1.40 Oil), and a hybrid or PMT detector. Scaling and merging of the fluorescence images were done using ImageJ, Fiji. For 3D image, z-stacked images were considered at the pitch of 1.0 µm and reconstructed using ImageJ, Fiji.

Refractive Index Measurement

Holographic visual microscospy measurements were made using commercially available Total Holographic Characteriaztion® instrument, xSight (Spheryx Inc., NY, USA).[2] xSight is the holographic analysis type particle counter analyzer, which can measure particle size and refractive index of the individual particle (size up to 10 µm) using the comparative analysis between measured inference pattern (hologram) and from scattering theory (Lorentz-Mie theory).[2-4] Coacervate samples for PEG-P(Asp-*Pr-OH*), σ=0.46 and PEG-P(Asp-Bu), σ=0.52 without protein was prepared in 25 mM HEPES buffer, 0 mM NaCl, pH 7.4 as the method described above. Special microfluidic measurement cell (Spheryx Cell) was used where 30 µL of dispersed coacervate sample was flowed. Obtained data of RI values for different particle size were further cleaned by selecting the particle that showed only spherical morphology (symmetry) and uncertainty in diameter (d) measurement < 0.05 µm. Then, RI values were plotted against particle size for each coacervate sample. Average value of refractive index (RI) of all size range was used for comparison.

3. Polypaptide Synthesis and Characterization

Polyethylene glycol-block-poly(β-benzyl-L-aspartate) (PEG-PBLA)—PEG-PBLA was synthesized via ring opening polymerization using β-benzyl-L-aspartate N-carboxy-anhydride (BLA-NCA) as a monomer and MeO-PEG-NH_2 as a macroinitiator.[5] 0.134 mmol of MeO-PEG-NH_2 (average MW 2234) was dissolved in Benzene in a Schlenk flask and freeze-dried overnight in vacuo (< 50 Pa, -50 °C). A freeze-dried sample was dissolved in super dehydrated DMF with N_2 purging. 1.15 equivalent of BLA-NCA (Mw 249.69) to PEG primary amines was measured in an N_2-glove box in a separate Schlenk flask and dissolved in super dehydrated DCM and super dehydrated DMF under the nitrogen atmosphere such that ratio of DCM and DMF is 2:1 (v/v) in the final reaction mixture. Then, the resulting solution of monomer was rapidly added to the initiator solution under the nitrogen gas stream using a pre-dried

This journal is © The Royal Society of Chemistry 20xx

Please do not adjust margins

Please do not adjust margins

syringe. The reaction was continually stirred at 40 °C for 48 h in the dark. The white turbid solution was then dissolved in a limited amount of super dehydrated $CHCl_3$ thus obtaining a clear solution. The polymer was recovered after precipitation in hexane: ethyl acetate (6:4 v/v) solution and vacuum filtered using a PTFE membrane filter. The retrieved polymer was re-dissolved in a limited amount of $CHCl_3$ and freeze-dried using excess benzene as azeotrope for 48 h. (Yield = 79%). The degree of polymerization (DP) of the BLA segment was determined to be 85 based on the peak intensity ratio of the protons of the PBLA side chains 'e' (phenyl proton, δ 7.27-7.23 (m, 424H)) to the methylene protons in the PEG 'a' (OCH_2CH_2, δ 3.51(s, 196H)) and aspartate unit 'c' and 'd' (-CO-CH-NH-, δ 2.58, 2.82 (m, 81-83H) ; -CO-CH_2-CH-, δ 5.02(d, J=13.3 Hz, H159)) segments, respectively (Figure S1).

Polyethylene glycol-block-poly-L-aspartate (PEG-PAsp)— PEG-PAsp was obtained via direct hydrolysis of PEG-PBLA according to the method given in a previous report (Scheme S1).[6] Typically, ~200 mg PEG-PBLA was dissolved in 5 ml acetonitrile, followed by the addition of 0.5M $NaOH_{aq}$. (5 equiv. to total side chain of PBLA). The solution was stirred at room temperature for 1 h in an amber glass vial for complete hydrolysis. Then, the resulting clear solution was dialyzed with a dialysis membrane (MWCO 3,500) by ultrapure water (UPW) for 48 h. The final product, PEG-PAsp, was collected by subsequent freeze drying (Yield ~90%). ^{1}H NMR measurement was carried out in D_2O at 80 °C to determine the DP of the PAsp segment. The DP of the PAsp segment was calculated to be 82 from the peak integral ratio of the methylene protons of PEG (OCH2CH2; δ 3.70; 196H) and PAsp 'b', and 'c' (CHCH2COO; δ 2.8 and 4.50-4.70; 164H and 81H, respectively) (Figure S2). SEC measurement was performed in 10 mM PB (pH 7.4) containing 500 mM NaCl (Figure S12a).

This journal is © The Royal Society of Chemistry 20xx

Please do not adjust margins

Please do not adjust margins

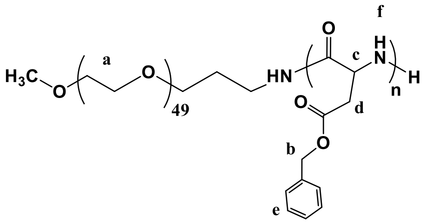

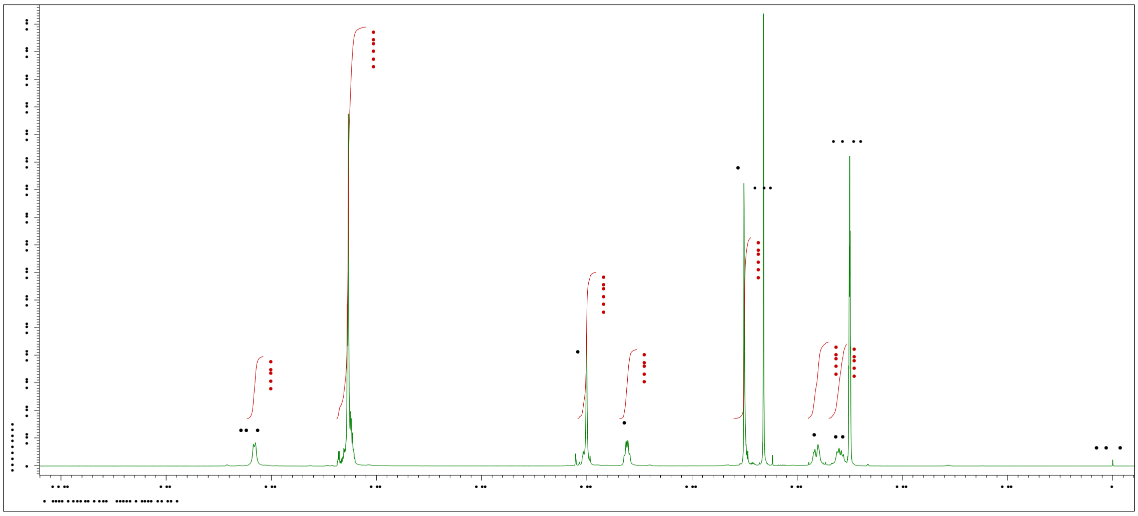

Fig. S1. ^1H NMR spectrum of PEG-PBLA in DMSO-d_6 at 25 °C.

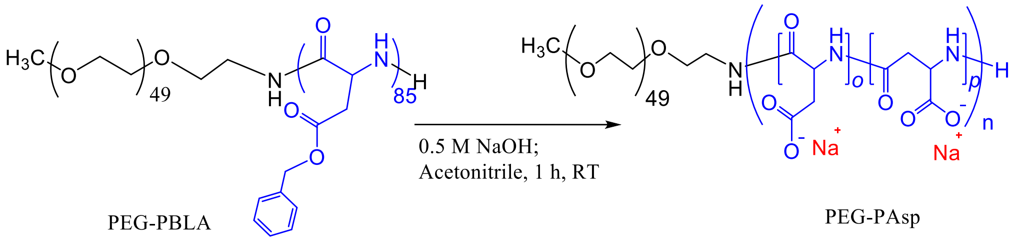

Scheme S1. Synthetic scheme for hydrolysis of PEG-PBLA to get PEG-PAsp

Please do not adjust margins

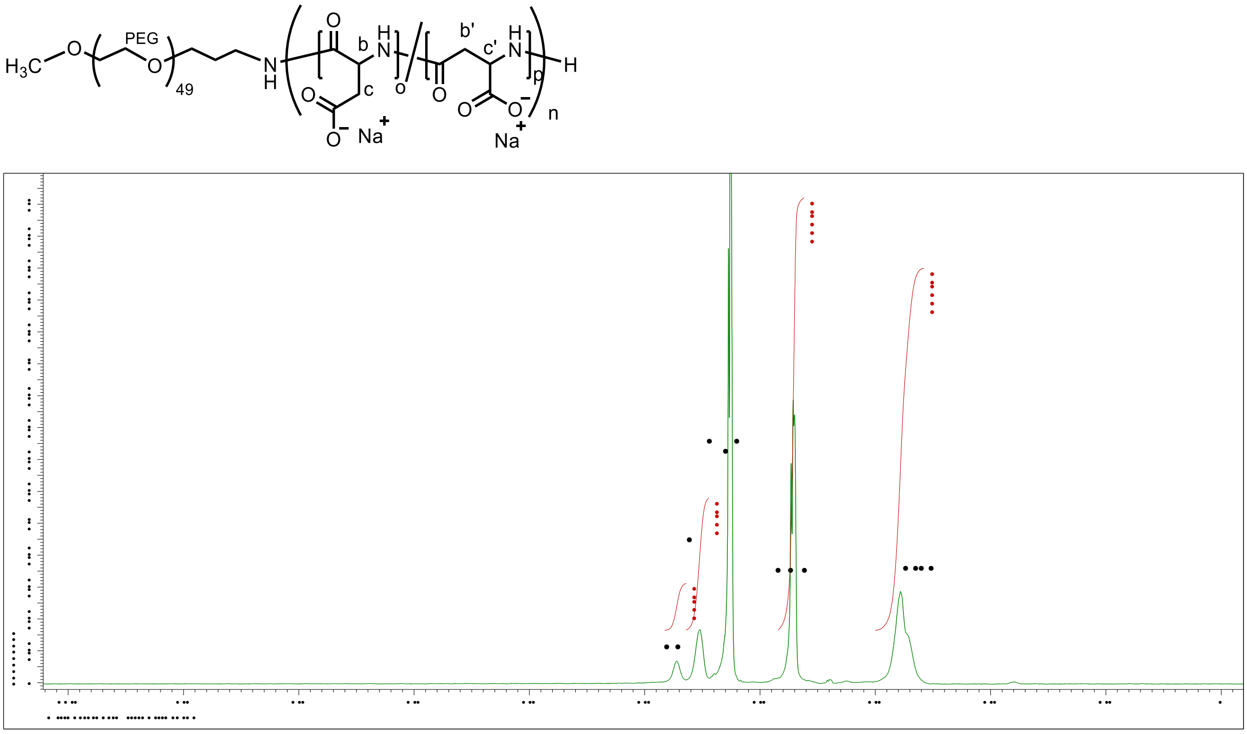

Fig. S2. ^{1}H NMR of PEG-PAsp σ=1.0 (Scans=64) in D_2O, 400 MHz, 80 °C.

Polyethylene glycol-block-poly(3-hydroxypropyl-α,β-aspartamide) (PEG-P(Asp-*Pr-OH*))—Pendant hydroxyl groups were attached to the side chain of PAsp via partial aminolysis of the PEG-PBLA using 3-amino-propan-1-ol (NH_2-*Pr-OH*) according to the previously reported method with a minor modification (Scheme S2).[5] Briefly, PEG-PBLA was dissolved in dry DMF at the concentration of ~10 mg/mL. Then, to obtain the polymer with a different modification rate, varied equivalents of NH_2-*Pr-OH* (to the number of side chains of PBLA; Table S1) was added under the N_2 atmosphere. Subsequently, 0.3 eq. equivalent of DIPEA was added as a catalyst. The reaction mixture was stirred at room temperature for 3 h and followed by re-precipitation using diethyl ether as a poor solvent. The resulting white precipitate was collected by suction filtration and dried in vacuo. The collect-ed solid was treated with 0.5 M $NaOH_{aq}$. for 1 h to remove benzyl groups by hydrolysis. Then the clear solution was dialyzed with dialysis membrane (MWCO 3,500) by UPW for 48 h and recovered via freeze-drying (Yield ~ 85%). The DP was determined from the ratio of peak integral of ^{1}H NMR spectra of the methylene protons of PEG (-OCH_2CH_2-; δ 3.70) and PAsp ($CHCH_2COO$; δ 2.75 and 4.50-4.70; peaks b and c in Fig. S2), whereas the number of modified residues per polymer chain (m) was computed from the peak integral ratio of the methylene protons of PEG (OCH_2CH_2; δ 3.70) and methylene protons of the side chain (-$CH_2CH_2CH_2OH$; δ 1.85, 3.2 and 3.6; peaks e, d, and f in Figs. S3-S7). SEC measurements were performed in 10 mM PB (pH 7.4) containing 500 mM NaCl (Figure S12b-f).

σ=0.84: (DP=83, m=13) 1H NMR (400 MHz, D_2O) δ 4.59-4.84 (27H), 4.44-4.59 (48H), 3.65-3.83 (196H), 3.48-3.64 (33H), 3.10-3.35 (28H), 2.50-3.02 (165H), 1.62-1.85 (23H)

σ=0.74: (DP=80, m=21) 1H NMR (400 MHz, D_2O) δ 4.60-4.85 (18H), 4.45-4.58 (45H), 3.66-3.96 (196H), 3.15-3.45 (42H), 2.53-3.00 (160H), 1.61-1.95 (42H)

σ=0.69: (DP=78, m=24) 1H NMR (400 MHz, D_2O) δ 4.45-4.83 (98H), 3.64-3.97 (196H), 3.51-3.64 (42H), 3.16-3.34 (48H), 2.59-3.06 (156H), 1.67-1.86 (48H).

σ=0.61: (DP=74, m=29) 1H NMR (400 MHz, D_2O) δ 4.58-4.85 (38H), 4.41-4.58 (34H), 3.64-3.97 (196H), 3.48-3.64 (56H), 3.12-3.36 (60H), 2.51-3.04 (148H), 1.60-1.88(56H)

σ=0.46: (DP=85, m=46) 1H NMR (400 MHz, D_2O) δ 4.58-4.85 (48H), 4.42-4.58 (29H), 3.66-3.82 (196H), 3.49-3.66 (98H), 3.16-3.35 (92H), 2.49-3.01 (170H), 1.65-1.84 (92H)

 This journal is © The Royal Society of Chemistry 20xx

Please do not adjust margins

Table S1. Reaction condition for partial aminolysis for PEG-P(Asp-*Pr-OH*)

Sample	Equiv. of NH_2-*Pr-OH* (x)	DIPEA equiv.	Reaction Time (hours)	Temperature
~20% mod.	0.8	0.3	3	r.t
~30% mod.	1.0~1.2	0.3	3	r.t
~40% mod.	1.8	0.5	3	r.t
>50% mod.	3	0.5	3	r.t

r.t: Room temperature (22-26 °C)

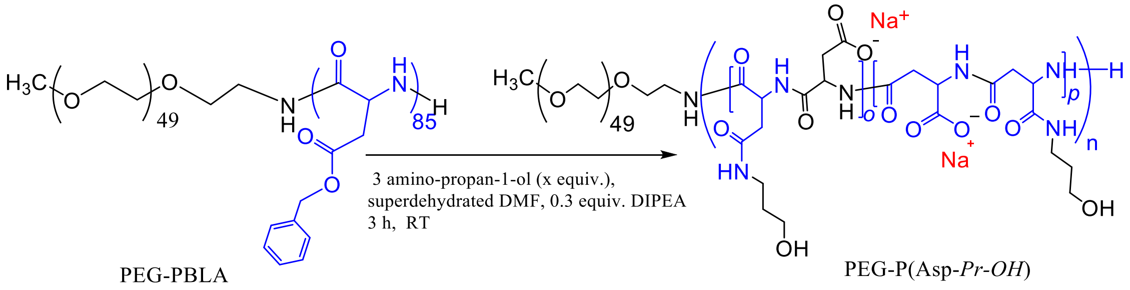

Scheme S2. Synthetic scheme for partial aminolysis of PEG-PBLA to get PEG-P(Asp-*Pr-OH*)

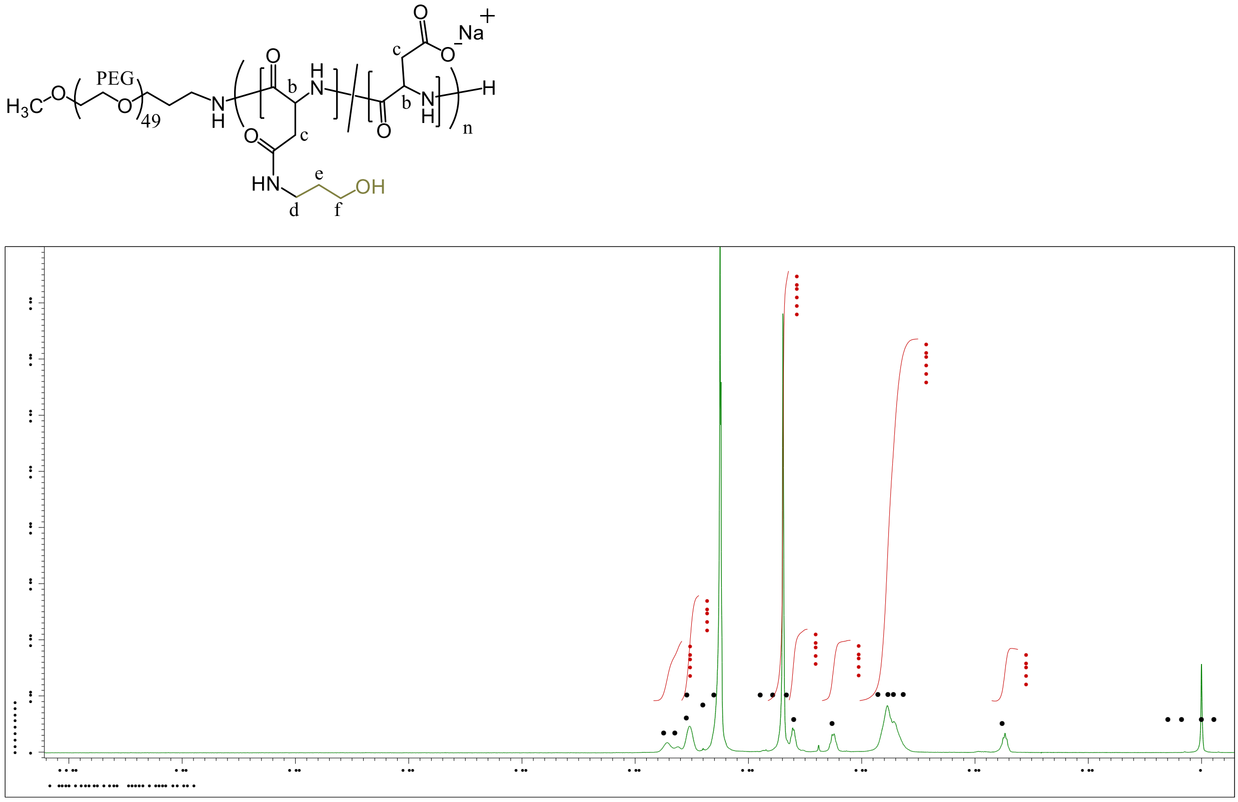

Fig. S3. ^{1}H NMR of PEG-P(Asp-*Pr-OH*), σ= 0.84 (Scans=64) in D_2O, 400 MHz, 80 °C, TMSP as internal reference.

Please do not adjust margins

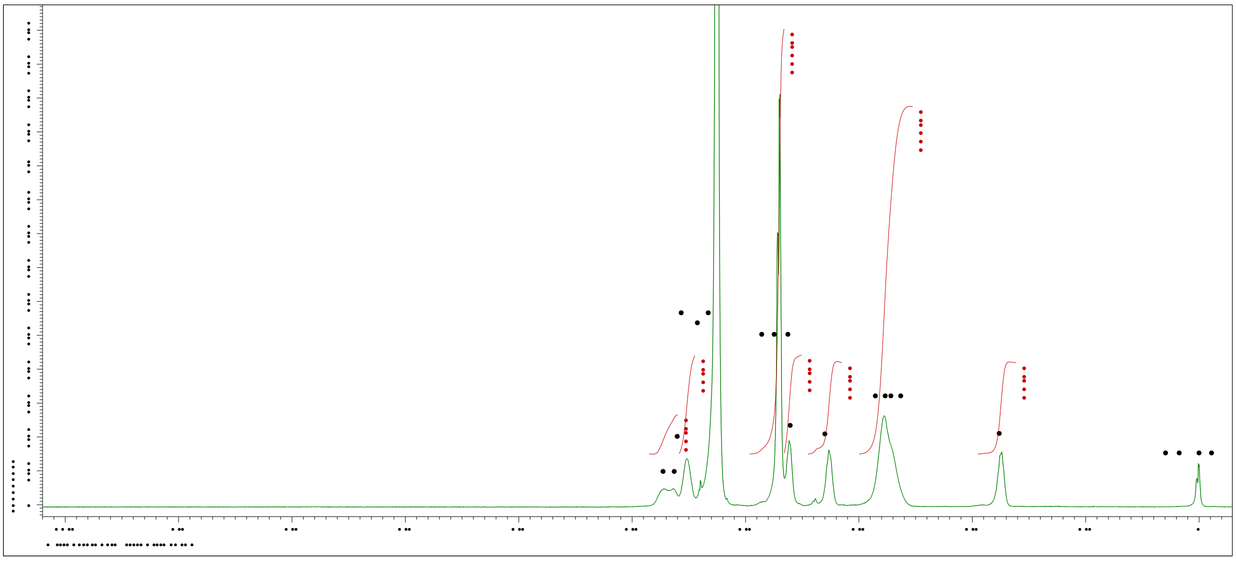

Fig. S4. ^{1}H NMR of PEG-P(Asp-*Pr-OH*), σ = 0.74 (Scans = 64) in D_2O, 400 MHz, 80 °C, TMSP as internal reference.

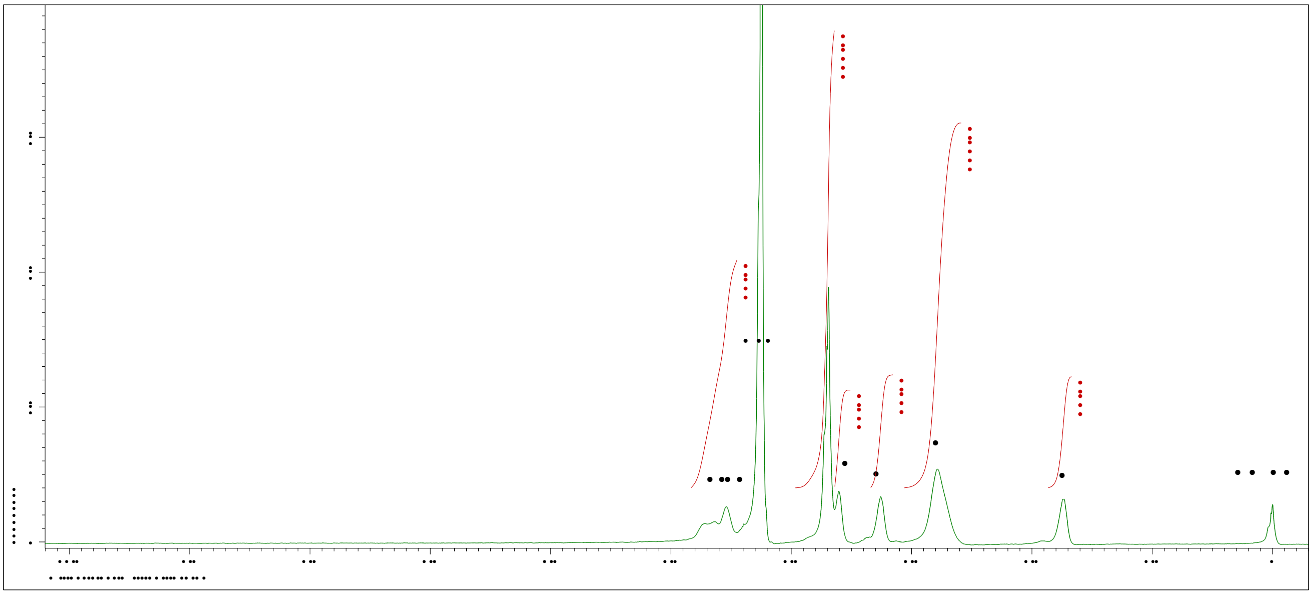

Fig. S5. ^{1}H NMR of PEG-P(Asp-*Pr-OH*), σ = 0.69 (Scans = 64) in D_2O, 400 MHz, 80 °C, TMSP as internal reference.

This journal is © The Royal Society of Chemistry 20xx

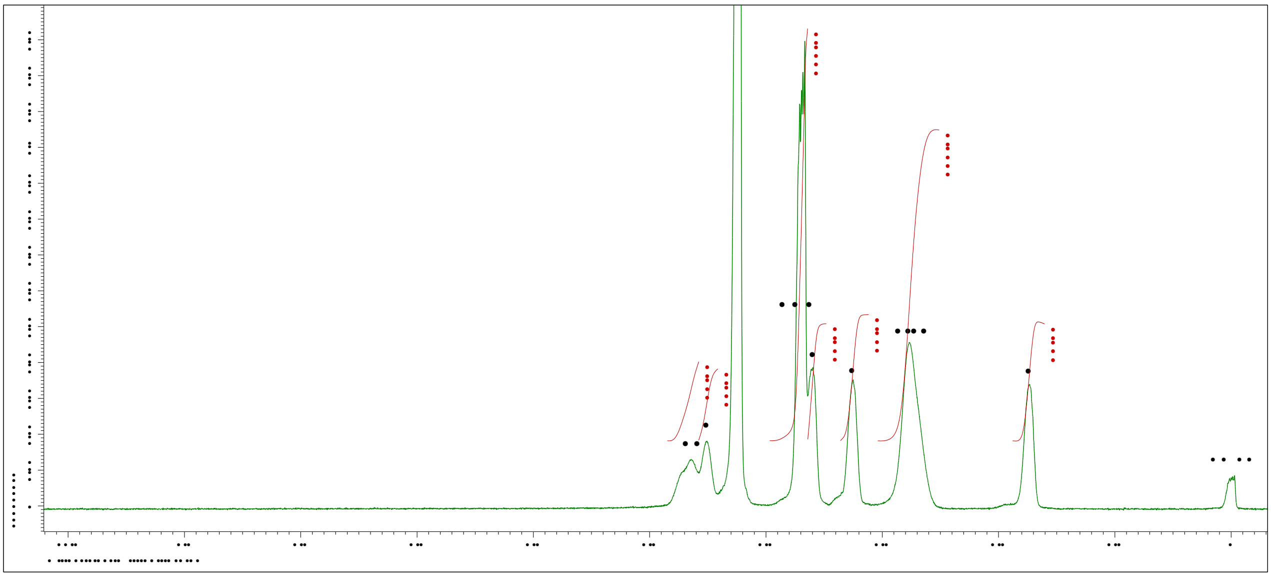

Fig. S6. ^{1}H NMR of PEG-P(Asp-*Pr-OH*), σ= 0.61 (Scans=64) in D_2O, 400 MHz, 80 °C, TMSP as internal reference

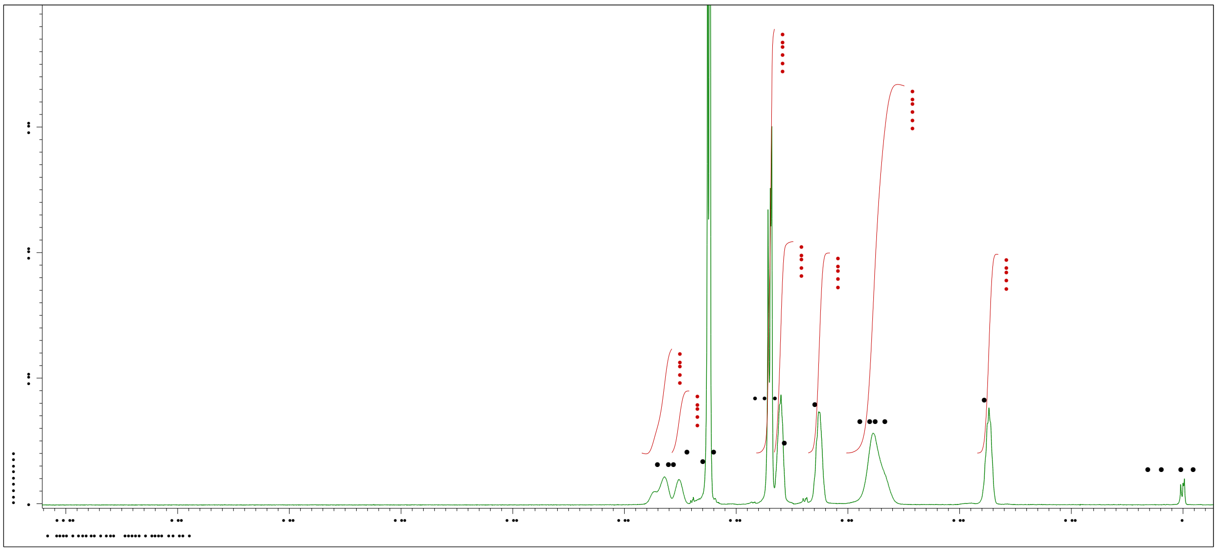

Fig. S7. ^{1}H NMR of PEG-P(Asp-*Pr-OH*), σ= 0.46 (Scans=64) in D_2O, 400 MHz, 80 °C, TMSP as internal reference.

Please do not adjust margins

Polyethylene glycol-block-poly(butyl-α,β-aspartamide) (PEG-P(Asp-*Bu*))— Pendant butyl group were introduced to the aspartate side chains via partial aminolysis of the PEG-PBLA using n-butyl amine according to the previously reported method with a minor modification (Scheme S3).[5] PEG-PBLA was dissolved in dry DMF at the concentration of ~10 mg/mL. Then, to obtain the polymer with different modification rates, varied equivalents of n-butyl amine (w.r.t the number of side chains of PBLA; Table S2) was added under N_2 atmosphere. Subsequently, 0.5 eq. of DIPEA was added as a catalyst. The reaction was conducted at room temperature for 5.5 h, and the reaction mixture was subjected to re-precipitation using a mixture of hexane and ethyl acetate (6:4 v/v) as a poor solvent. The resulting white precipitate was collected by suction filtration and dried in vacuo. The collected white powder was subjected to hydrolysis to remove benzyl groups from side chains using 0.5 M $NaOH_{aq}$. The clear solution thus obtained was dialyzed with a dialysis membrane (MWCO 3,500) against UPW for 48 h, followed by freeze drying, to give PEG-P(Asp-*Bu*) (Yield ~90%). The DP was computed from the ratio of peak integral of ^{1}H NMR spectra of the methylene protons of PEG (-OCH_2CH_2-; δ 3.70) and PAsp (-$CHCH_2COO$-; δ 2.75 and 4.50-4.70; peaks b and c in Figs. S2), whereas the number of modified residues per polymer chain was computed from peak integral ratio of the methylene protons of PEG (-OCH_2CH_2-; δ 3.70) and methylene protons (-$CH_2CH_2CH_2$-; δ 1.3, 1.5 and 3.2; peaks e, d, and f in Figs. S8-11) and methyl proton (-CH_3; δ 0.85; peak g in Figs. S8-11) of the side chains. SEC measurements were performed in 10 mM PB (pH 7.4) containing 500 mM NaCl (Figure S13).

σ=0.87 ^{1}H NMR (400 MHz, D_2O) δ 4.64-4.88 (30H), 4.45-4.63 (59H), 3.60-3.88 (196H), 3.06-3.32 (30H), 2.55-3.02 (165H), 1.40-1.58 (23H), 1.23-1.40 (21H), 0.83-1.02 (35H)
σ=0.70 ^{1}H NMR (400 MHz, D_2O) δ 4.43-4.86 (84H), 3.49-3.98 (196H), 3.04-3.32 (52H), 2.54-3.02 (163H), 1.37-1.64 (50H), 1.20-1.37 (44H), 0.79-1.04 (69H)
σ=0.65 ^{1}H NMR (400 MHz, D_2O) δ 4.43-4.89 (87H), 3.58-3.86 (196H), 3.04-3.34 (66H), 2.52-3.03 (166H), 1.40-1.61 (60H), 1.21-1.40 (58H), 0.78-1.03 (88H)
σ=0.52 ^{1}H NMR (400 MHz, D_2O) δ 4.39-4.88 (80H), 3.58-3.87 (196H), 3.01-3.31 (82H), 2.50-2.96 (168H), 1.38-1.60 (83H), 1.14-1.37 (80H), 0.77-1.02 (123H)

Table S2. Reaction condition for partial aminolysis for PEG-P(Asp-Bu)

Sample	Eq. of butyl amine (X)	DIPEA eq.	Reaction Time (hours)	Temperature
~20% mod.	0.8	0.3	5.5	r.t
~30% mod.	1.0	0.3	5.5	r.t
~40% mod.	1.8	0.5	5.5	r.t
>50% mod.	3	0.5	5.5	r.t

r.t: Room temperature (22-26 °C)

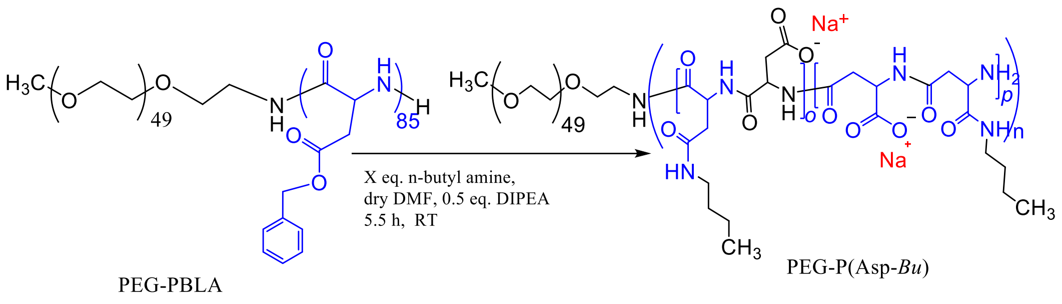

Scheme S3. Synthetic scheme for partial aminolysis of PEG-PBLA to get PEG-P(Asp-*Bu*)

This journal is © The Royal Society of Chemistry 20xx

Please do not adjust margins

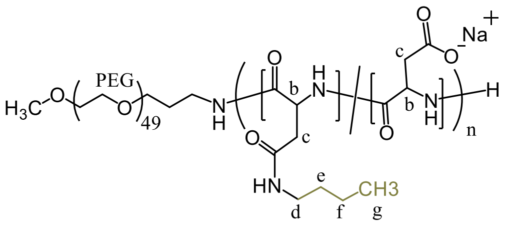

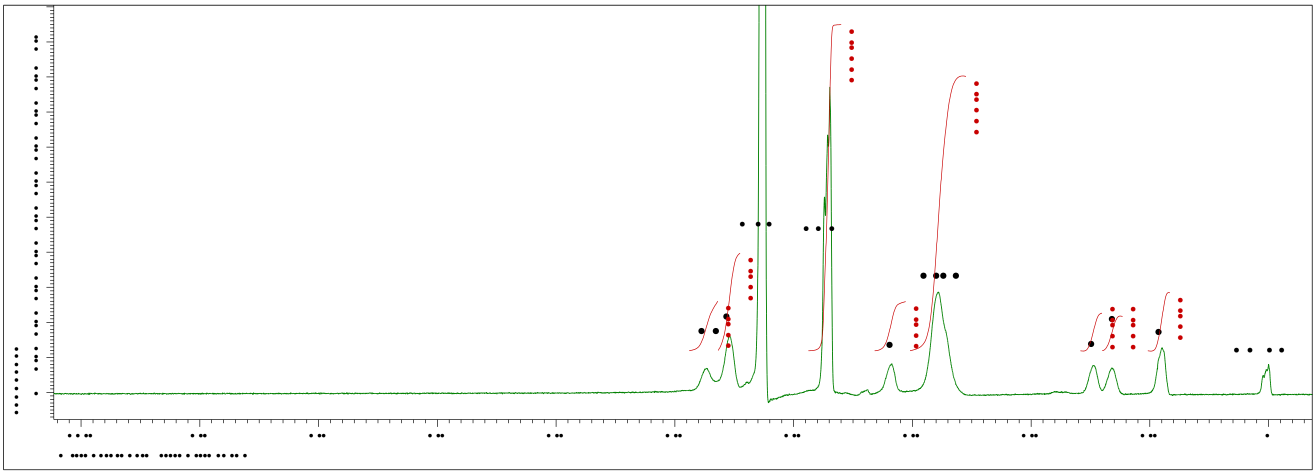

Fig. S8. ^{1}H NMR of PEG-P(Asp-*Bu*), σ= 0.87 (Scans=64) in D_2O, 400 MHz, 80 °C, TMSP as an internal reference.

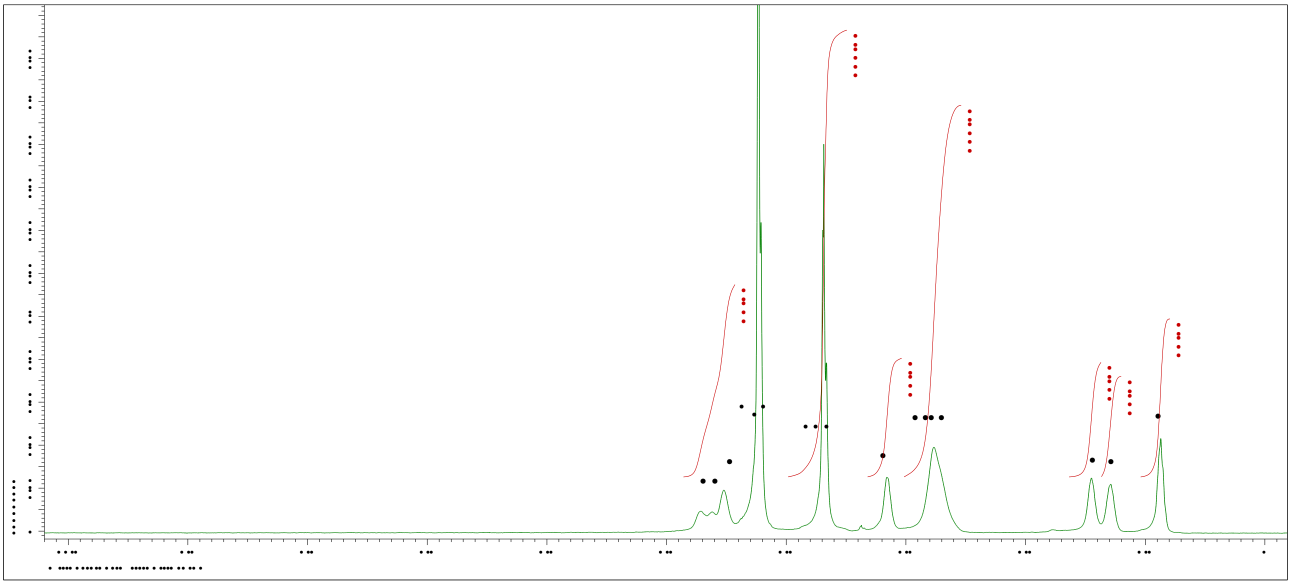

Fig. S9. ^{1}H NMR of PEG-P(Asp-*Bu*), σ= 0.70 (Scans=64) in D_2O, 400 MHz, 80 °C

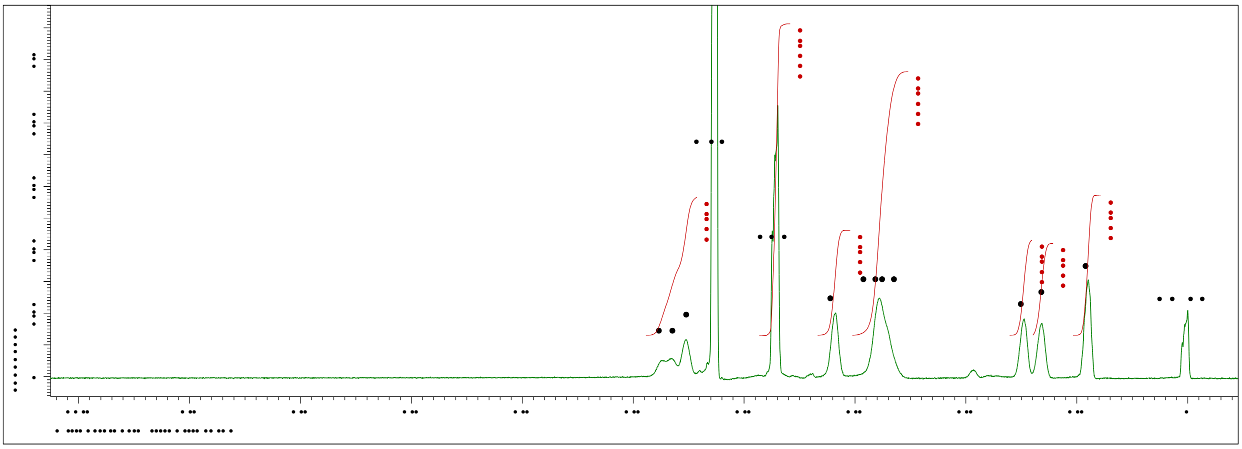

Fig. S10. ^{1}H NMR of PEG-P(Asp-*Bu*), σ= 0.65 (Scans=64) in D_2O, 400 MHz, 80 °C, TMSP as an internal reference.

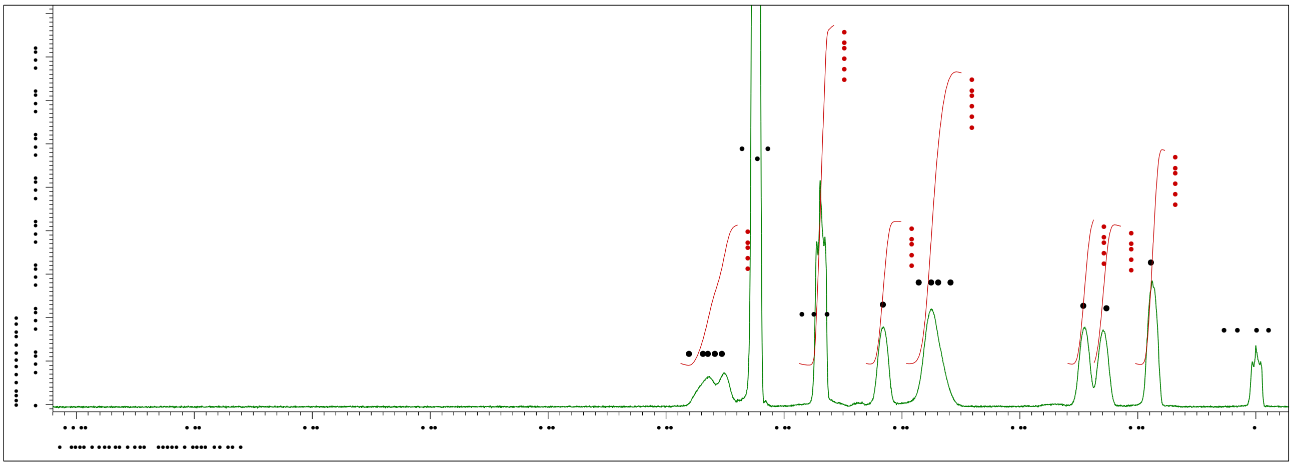

Fig. S11. ^{1}H NMR of PEG-P(Asp-*Bu*), σ= 0.52 (Scans=64) in D_2O, 400 MHz, 80 °C, TMSP as an internal reference.

This journal is © The Royal Society of Chemistry 20xx

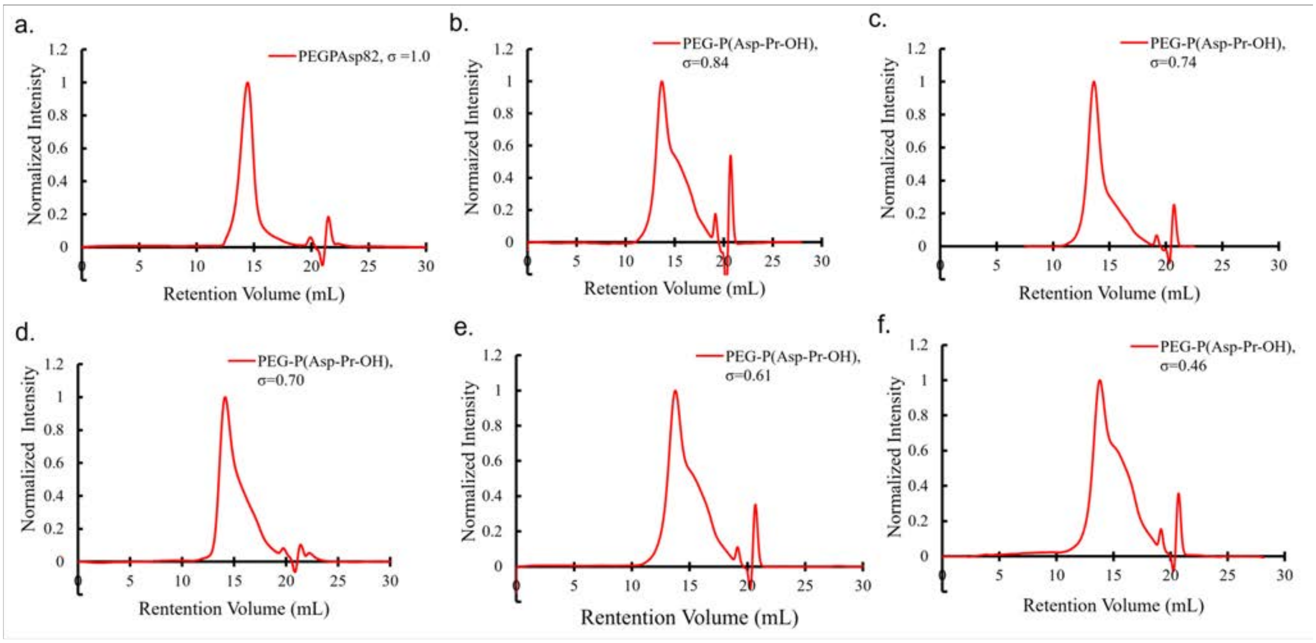

Fig. S12. Size-exclusion chromatograms of hydroxyl modified polyions a. PEG-PAsp, b. PEG-P(Asp-*Pr-OH*), σ = 0.84, c. PEG-P(Asp-*Pr-OH*), σ = 0.74, d. PEG-P(Asp-*Pr-OH),* σ = 0.70, e. PEG-P(Asp-*Pr-OH*), σ = 0.61, and f. PEG-P(Asp-*Pr-OH*), σ = 0.46

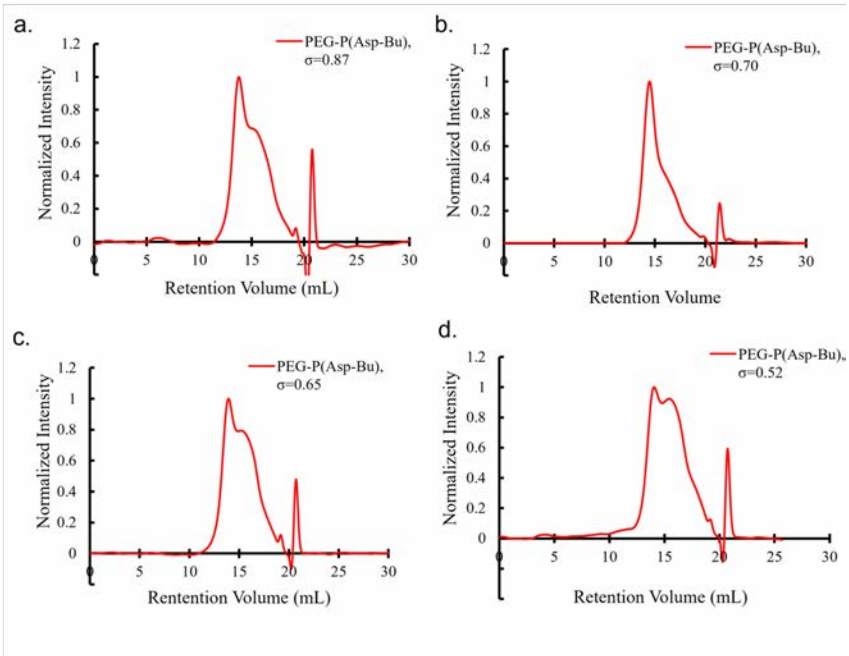

Fig. S13. Size-exclusion chromatograms of butyl modified polyions. a. PEG-P(Asp-*Bu*), σ=0.87, b. PEG-P(Asp-*Bu*), σ=0.70, c. PEG-P(Asp-*Bu*), σ=0.65, d. PEG-P(Asp-*Bu*), σ=0.52.

The broad shape of the chromatograms of the modified polymer analyzed via size-exclusion chromatography is partly because of chain degradation during aminolysis[7] but mostly because of the conformational difference in polymer ensemble, that is, the varying extent of interaction with the SEC column. Preparative SEC of the PEG-P(Asp-Pr-OH), σ =0.46, was performed using HiLoad 16/600 Superdex 75 pg column at flowrate of 1 ml/min using 10 mM PB, 500 mM NaCl (pH 7.4) as eluent at 10 mg/mL concentration (3 mL volume). Three fractions of SEC viz. 34-47, 48-53, and 54-68 were collected using an automatic dispenser system as shown in Figure S14a. Collected samples were freeze-dried and SEC profile and 1H NMR were taken for each sample. For each purified fraction, there were no significant differences in average degree of modification (Figure S14c). Therefore, samples without further purification were used.

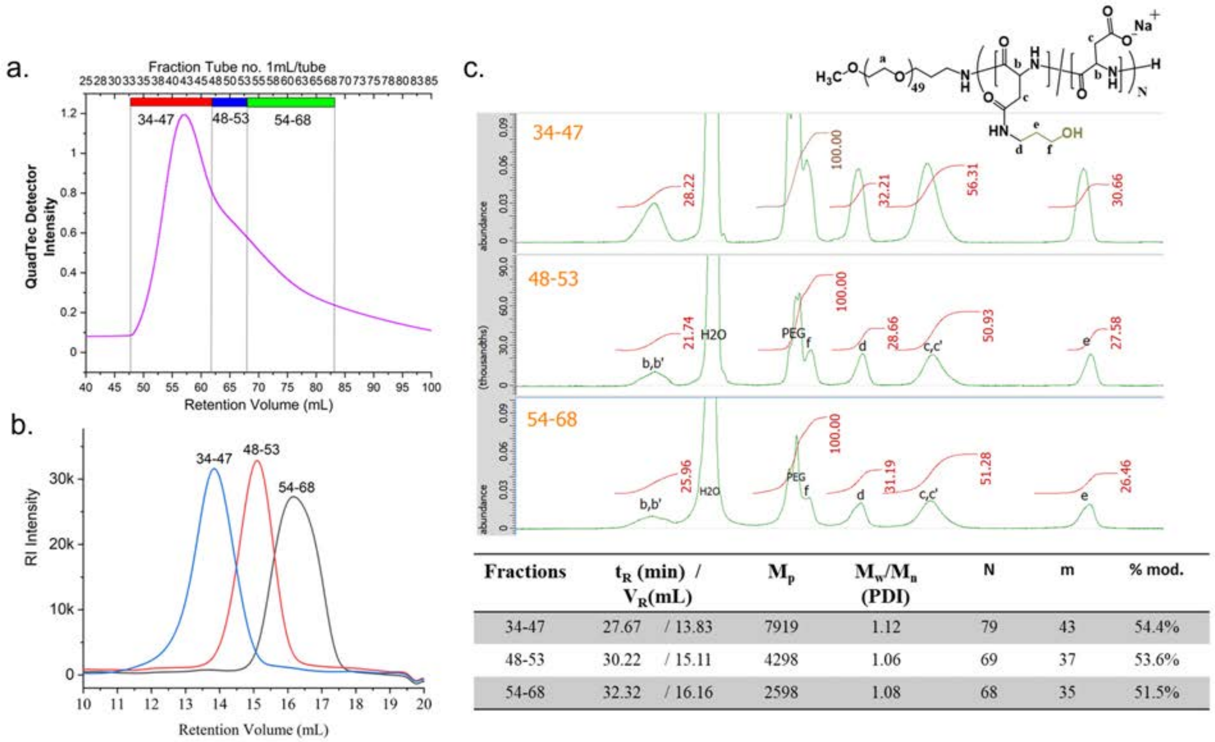

Fractions	t_R (min) / V_R(mL)	M_p	M_w/M_n (PDI)	N	m	% mod.
34-47	27.67 / 13.83	7919	1.12	79	43	54.4%
48-53	30.22 / 15.11	4298	1.06	69	37	53.6%
54-68	32.32 / 16.16	2598	1.08	68	35	51.5%

Fig. S14. a. SEC profile of PEG-P(Asp-*Pr-OH*), σ=0.46. Three fractions were collected for further characterization. b. SEC profiles of each fraction. c. ^{1}H NMR spectra of each fraction (top) indicating similar degree of modification (Table in the bottom).

This journal is © The Royal Society of Chemistry 20xx

Fluorescence labelling of polymers

For labelling polymers, ~10 mg/mL polymer solution was prepared in 100 mM PB buffer, pH 8.0, and subsequently, 1 eq. of activated fluorescence probe (NHS rhodamine or Cy3-NHS) was added. The mixture was stirred for 24 h, at room temperature. The polymer was purified via dialysis using membrane of MWCO of 3,500, for first 2-3 times exchange in 5% DMSO (for Cy3 only) and then in UPW (5-6 times exchange), until the outside solution become colorless. The purified polymer solution freeze-dried. Labelling ratio was determined by UV-vis spectroscopy using a spectrophotometer JASCO V-670 (Jasco International Co., Ltd., Tokyo, Japan) by dissolving measured weight in 25 mM HEPES buffer to make 5 mg/mL and making appropriate dilution.

4. Protein Expression and Purification:

Preparation of GFPs

Sequences:
> Enhanced_GFP(EGFP)
ATGGTAAGTAAGGGGGAAGAGTTGTTTACAGGCGTTGTACCGATTTTAGTAGAACTGGATGGTGACGTGAACGGGCATAAGTTCAGTGTCTC
CGGTGAGGGTGAGGGCGACGCGACCTATGGAAAATTAACACTGAAATTTATCTGCACTACCGGGAAACTTCCCGTACCATGGCCCACGCTTGT
AACAACGCTTACATATGGAGTGCAATGTTTTTCGCGGTACCCAGATCATATGAAGCAGCATGATTTTTTTAAGAGTGCTATGCCGGAGGGTTAC
GTGCAAGAGCGTACTATATTCTTCAAAGATGATGGTAACTACAAGACGCGTGCCGAAGTGAAATTCGAAGGAGATACACTTGTAAACCGCATT
GAGTTAAAAGGAATAGACTTCAAGGAAGACGGGAACATTCTGGGGCACAAATTGGAATATAACTATAACAGTCACAACGTTTACATTATGGCT
GATAAGCAAAAAAACGGCATTAAAGTAAATTTTAAAATCCGCCATAACATAGAAGACGGATCGGTCCAGTTAGCCGACCACTATCAGCAAAAC
ACGCCGATAGGAGACGGCCCAGTGCTGTTGCCCGATAACCATTATCTGTCTACACAATCAGCCCTTTCAAAGGACCCCAACGAAAAGCGGGAT
CACATGGTCCTTTTGGAGTTCGTTACTGCTGCAGGAATAACCTTAGGTATGGACGAATTATACAAA

>GFP(+33)
ATGGCTAGCAAAGGAGAACGTCTCTTCCGTGGAAAAGTCCCAATTCTTGTTGAATTAAAAGGTGATGTTAATGGGCACAAATTTTCTGTCCGCG
GAAAAGGTAAAGGTGATGCAACAAACGGAAAACTTACCCTTAAATTTATTTGCACTACTGGAAAACTACCTGTTCCATGGCCAACACTTGTCAC
TACTCTGACGTATGGTGTTCAATGCTTTTCAAGATACCCGAAACACATGAAACGGCATGACTTTTTCAAGAGTGCCATGCCCAAAGGTTATGTA
CAGGAAAGAACTATATCGTTCAAAAAAGACGGGACCTACAAGACACGTGCTGAAGTCAAGTTTGAAGGTCGCACCCTTGTTAATAGAATCCGT
TTAAAAGGTCGTGATTTTAAAGAAAAAGGAAACATTCTTGGACACAAATTGCGCTACAACTTTAACTCACACAAAGTATACATCACCGCAGACA
AACGTAAGAATGGAATCAAAGCGAAATTCAAAATTAGACACAACGTGAAAGATGGAAGCGTTCAACTAGCAGACCATTATCAACAAAATACTC
CAATTGGCCGTGGCCCTGTCCTTTTACCACGTAACCATTACCTGTCCACACGCTCTGTGCTTTCGAAAGATCCCAAAGAAAAGAGAGACCACAT
GGTCCTTCTTGAGTTTGTAACAGCTGCTGGGATTAAACATGGCCGTGATGAACGTTACAAATAA

>GFP(-29)
ATGGCTAGCAAAGGAGAAGAACTCTTCGATGGAGTTGTCCCAATTCTTGTTGAATTAGATGGTGATGTTAATGGGCACGAATTTTCTGTCCGCG
GAGAGGGTGAAGGTGATGCAACAAACGGAGAACTTACCCTTAAATTTATTTGCACTACTGGAGAACTACCTGTTCCATGGCCAACACTTGTCAC
TACTCTGACGTATGGTGTTCAATGCTTTTCAGATTACCCGGATCACATGGATCAGCATGACTTTTTCAAGAGTGCCATGCCCGAAGGTTATGTAC
AGGAAAGAACTATATCGTTCAAAGATGACGGGACCTACAAGACACGTGCTGAAGTCAAGTTTGAAGGTGATACCCTTGTTAATAGAATCGAGT
TAAAAGGTATTGATTTTAAAGAAGATGGAAACATTCTTGGACACAAATTGGAATACAACTTTAACTCACACGATGTATACATCACCGCAGACAA
ACAAGAAAATGGAATCAAAGCGGAATTCGAAATTAGACACAACGTGGAAGATGGAAGCGTTCAACTAGCAGACCATTATCAACAAAATACTCC
AATTGGCGATGGCCCTGTCCTTTTACCAGACGATCATTACCTGTCCACAGAATCTGTGCTTTCGAAAGATCCCAACGAAGATAGAGACCACATG
GTCCTTCTTGAGTTTGTAACAGCTGCTGGGATTGATCATGGCATGGATGAACTATACAAATAA

Protein Expression

First DNA sequences were amplified using PrimeSTAR Max (Takara Bio Inc., Shiga, Japan) followed by agarose gel electrophoresis. The appropriate gel band was cut, dissolved, and purified using spin column. Then in-fusion method was used to conjugate the DNA of interest to plasmid vector (pET22B+) using Takara Bio Infusion Kit. After that, the obtained plasmid with gene of interest was transfected to JM109 E. coli competent cell using Mix and Go transformation kit (Zymo Research, Irvine, CA, USA Catalogue no. T3001). After selecting the ampicillin resistance colony, the transformed bacteria were cultured in LB (Amp) broth for overnight at 37 °C, and cells were pelleted. Plasmid extraction was done by typical cell lysis method and purified using spin column. Extracted plasmid was send for sequencing for verification and then only used further. For the protein expression, BL21 (DE3) E. coli strain was transfected with purified plasmid using Inoue transformation method.[8] Expression of protein was induced by adding 1 mM IPTG in culture broth.

Protein Isolation and Purification

After IPTG induced culture overnight, cells were pelleted at 2500 g for 20 min, 4 °C. Then resuspended in PBS buffer (pH 7.4). Cell lysis was carried out using ultrasonication @40 Watt, 20 KHz, for two 6 min runs with 5 min interval. Then cell debris were separated by centrifugation (8,000 g, 20 min). Collected supernatant was filtered further with 0.45 µm and 0.22 µm filter. For GFP

Please do not adjust margins

isolation, his-tag purification as carried out in 20 mM Tris 500 mM NaCl (pH 7.4) buffer with 0.5 M imidazole as eluent for collecting fraction. Further purification was carried out by dialyzing in 25 mM HEPES, 150 mM NaCl. It has been suggested that for supercharged proteins, at low ionic strength they can form coprecipitate with impurities like nucleic acids, phospholipids etc.[9] Such precipitates were observed and were filtered out repeatedly (2-3) times after dialysis. SDS-PAGE and fluorescence spectral analysis was caried out to confirm and see the purity of the sample. For GFP(-29), further purification was done using size-exclusion chromatography using HiLoad 16/600 Superdex 75 pg column. Protein Quantification was carried out using BCA assay with BSA as standard.

Fluorescence labelling of proteins

For labelling proteins by fluorescence dye, standard protocol provided from the vendor was used. Briefly, to the solution of 4-5 mg/ml of protein in 100 mM PB buffer (pH 8.0), 1−2 eq. of fluorescence dye (NHS rhodamine or FITC or Cy3-NHS, Cy5-NHS, pre-dissolved in DMSO) were added in dark amber glass vial. The mixture was stirred for 1 h at room temperature for BSA, Lysozyme, IgG and for 2 h, 4 °C for β-galactosidase, glucose-oxidase, HRP and DaO. The fluorescent labelled proteins were purified using PD-10 column (Cytiva, UK) pre-equilibrated with 25 mM HEPES buffer, and then concentrated by ultrafiltration using a membrane (MWCO 10,000; Vivaspin® 20, Sartorius Stedim Ltd., Stonehouse, UK) at 3,000 G, for 10 min. Final concentration and labelling ratio were determined by UV-vis spectroscopy using spectrophotometer JASCO V-670 (JASCO International Co., Ltd., Tokyo, Japan) and Fluorescence Emission Spectroscopy using the plate reader (Infinite Pro200, TECAN M PLEX, Tecan Japan Co. Ltd., Osaka, Japan) using standard calibration curve method using BSA as standard. The stocks of ~2 mg/mL were stored in -80 °C in aliquots after flash freezing in liquid N_2.

This journal is © The Royal Society of Chemistry 20xx

Please do not adjust margins

5. Supporting Results:

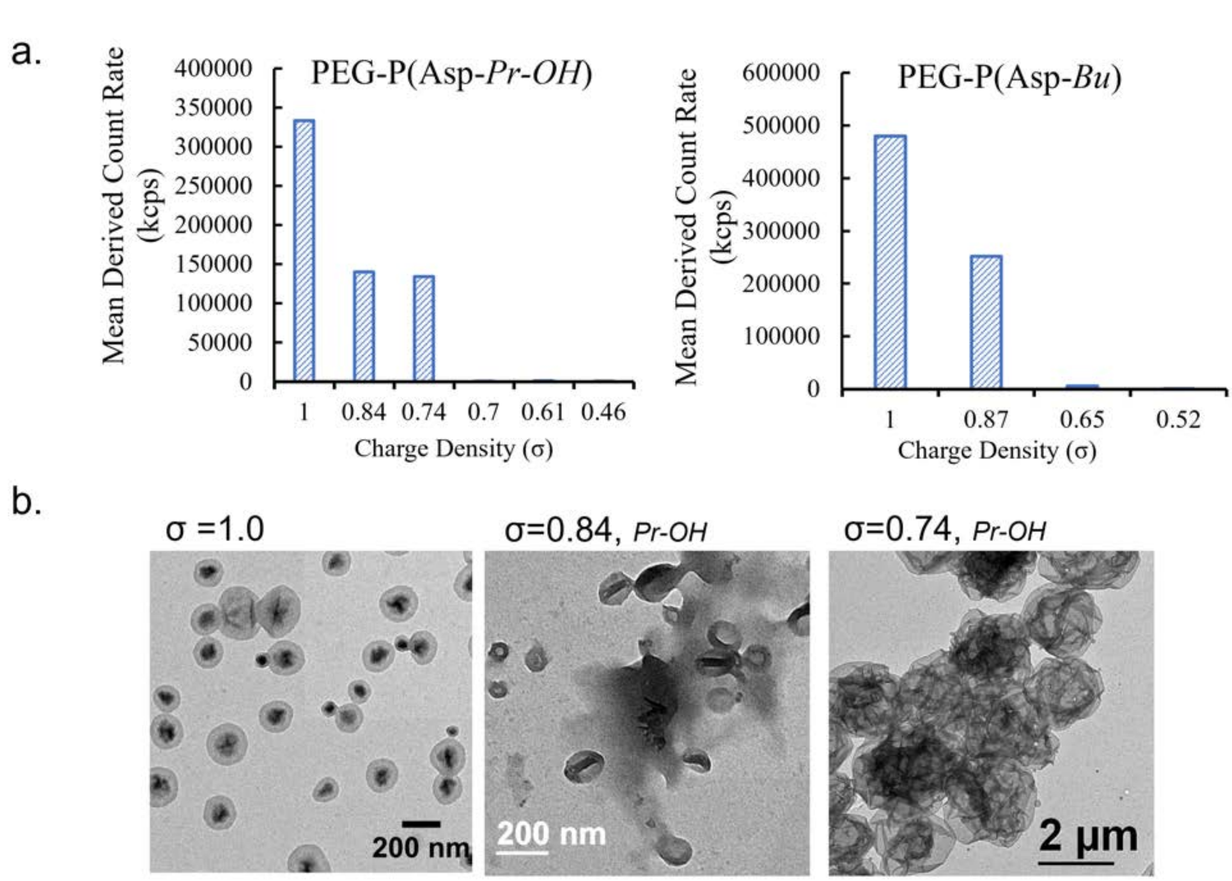

Fig. S15. a. Mean count rate in supernatant determined from dynamic light scattering for different σ of propanol and butyl modified samples at 0 mM NaCl showing nano-assemblies formed for σ > 0.7. b. Transmission electron micrographs of the vesicle formed at low ionic strength (0 mM NaCl) for σ > 0.7.

a.

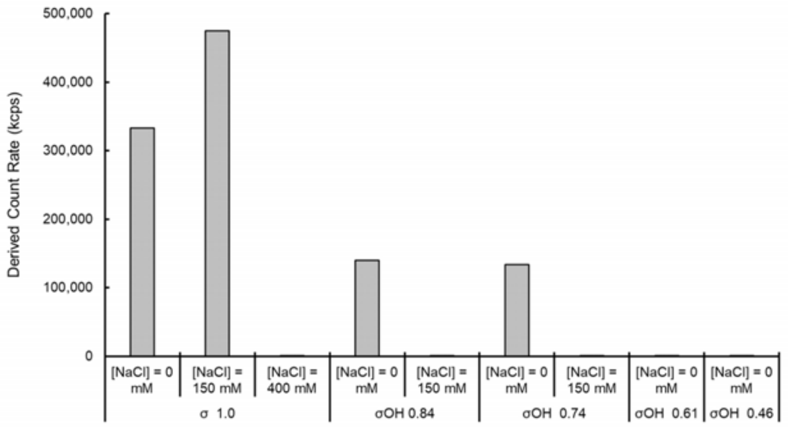

b.

σ	optimal [NaCl]
1	400 mM
0.84	150 mM
0.74	150 mM
0.61	0 mM
0.46	0 mM

Fig. S16. a. Mean count rate of the nano-assemblies determined by DLS in the supernatant of the polyion complex prepared at different NaCl concentration for PEG-P(Asp-*Pr-OH*) based complex coacervate. b. A list of optimal salt concentrations for different σ in coacervation determined by DLS measurement results shown in Figs. S15a and S16aand turbidity measurements shown in Fig. S17c.

 This journal is © The Royal Society of Chemistry 20xx

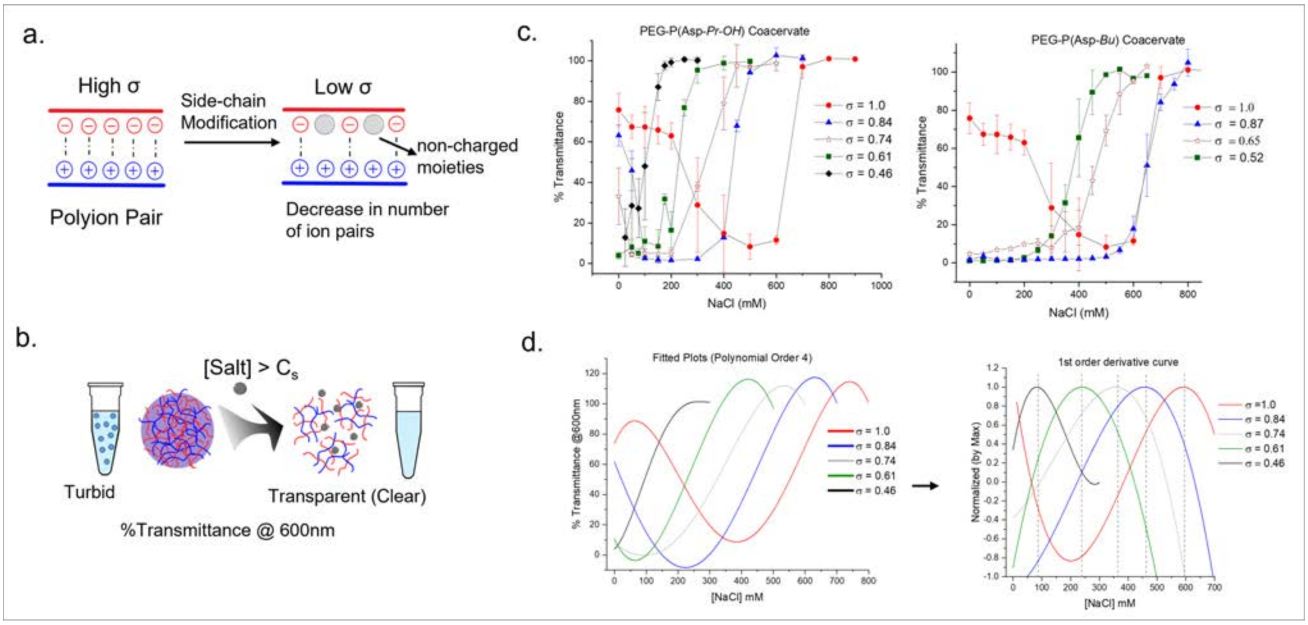

Fig. S17. a. Schematic diagram of side-chain modification by non-charged moieties and its impact on the number of ion-pairs in a polyion complex. b. Schematic illustration of the evaluation of critical salt concentrations by turbidity measurement using %Transmittance at 600 nm. c. Salt-concentration-dependency of %Transmittance of charge-reduced-polyion-based coacervate from PEG-P(Asp-*Pr-OH*) (left) and PEG-P(Asp-*Bu*) (right). d. Polynomial fittings and 1st order derivative curves obtained from %Transmittance curves to determine critical salt concentrations for various σ.

Please do not adjust margins

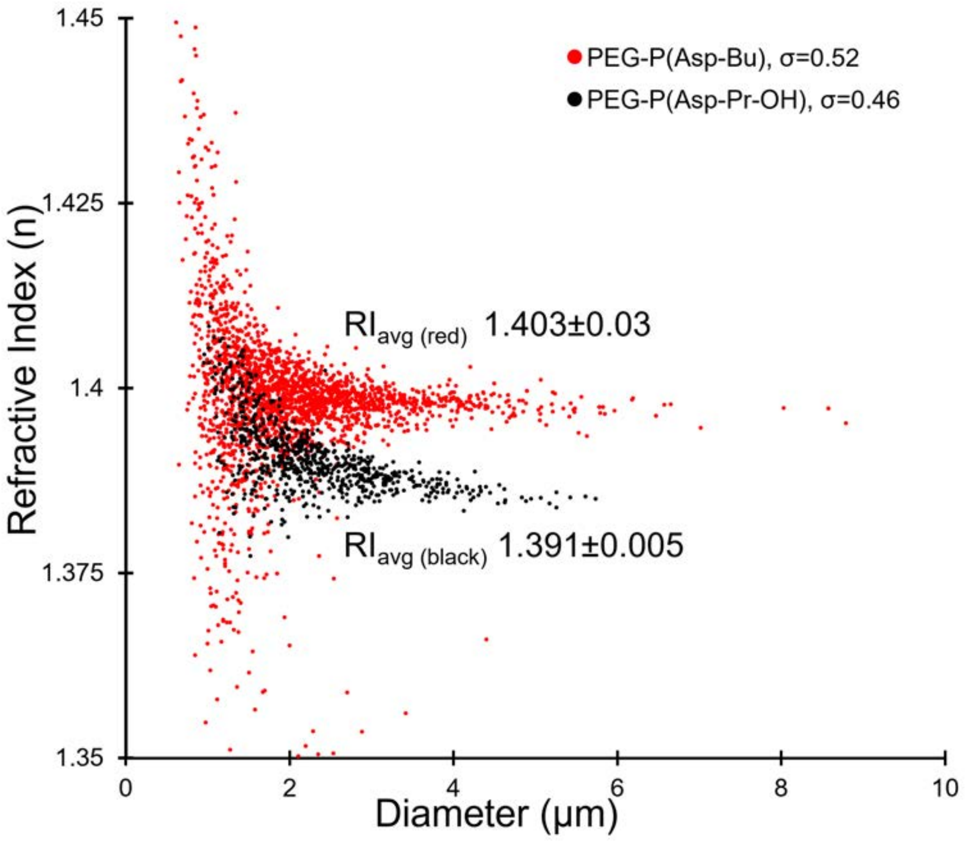

Fig. S18. Refractive indices (n) of coacervate droplets for different sizes ranging from 1 to 10 µm obtained using Xsight. [Coacervates prepared from PEG-P(Asp-*Bu*) (σ =0.52, red) and PEG-P(Asp-*Pr-OH*) (σ=0.46, black) at 25mM HEPES (pH 7.4, 0 mM NaCl).]

This journal is © The Royal Society of Chemistry 20xx

Please do not adjust margins

Please do not adjust margins

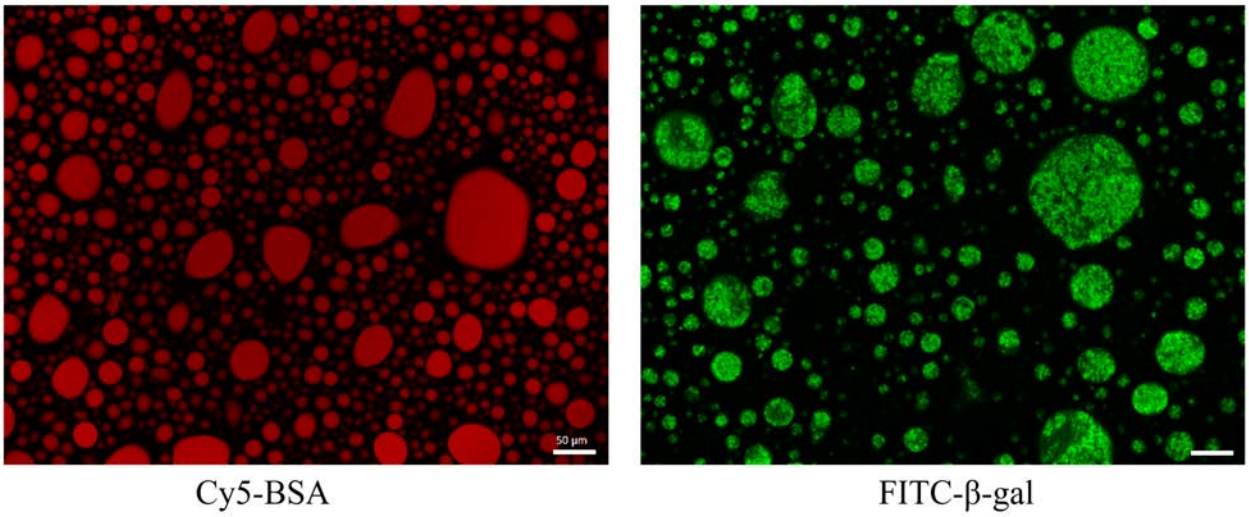

Fig. S19. Fluorescence image of coacervate droplets [σ = 0.61, PEG-P(Asp-*Pr-OH*)] prepared in the presence of Cy5-BSA (red) and fluorescein-β-gal (green). (Scale bars: 50 μm)

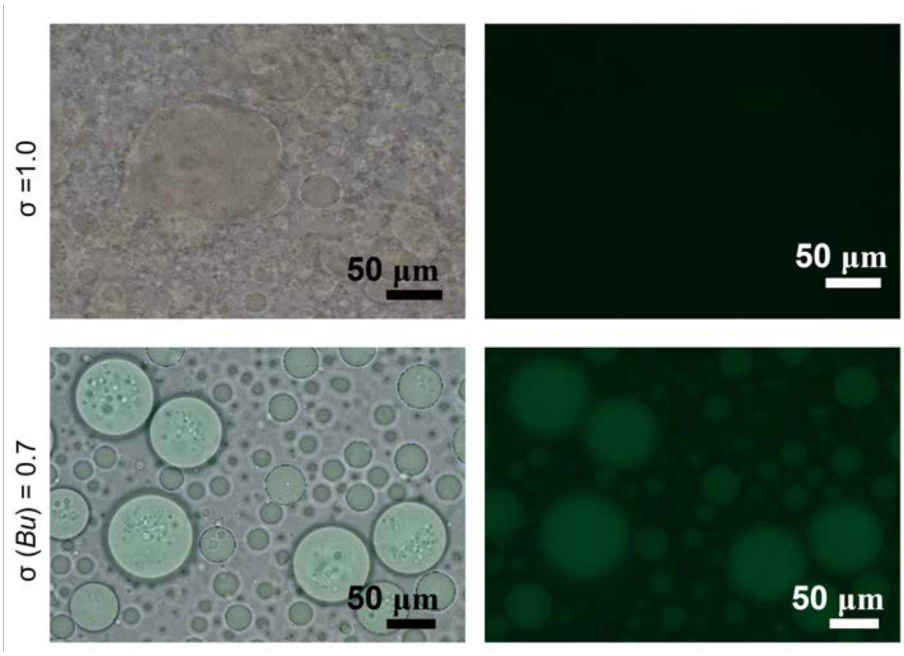

Fig. S20. Fluorescence and merged images of coacervate droplets prepared in the presence of FITC-BSA (green) and 50mM NaCl.

Please do not adjust margins

Please do not adjust margins

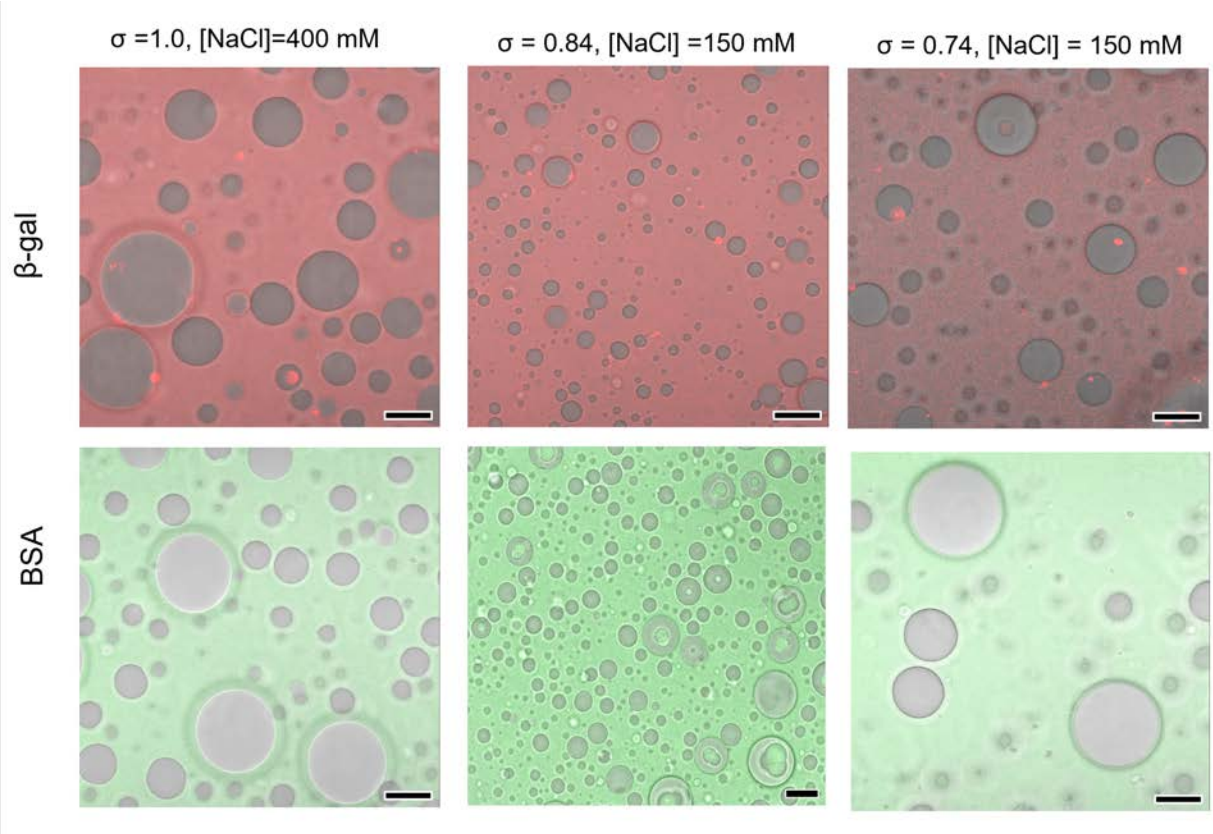

Fig. S21. Merged images of fluorescent and bright-field images of coacervate droplets prepared from PEG-P(Asp-*Pr-OH*) with high σ in the presence of rhodamine-labelled β-gal (red) and fluorescein-labelled BSA (green). (Scale bars = 20 µm)

This journal is © The Royal Society of Chemistry 20xx

Please do not adjust margins

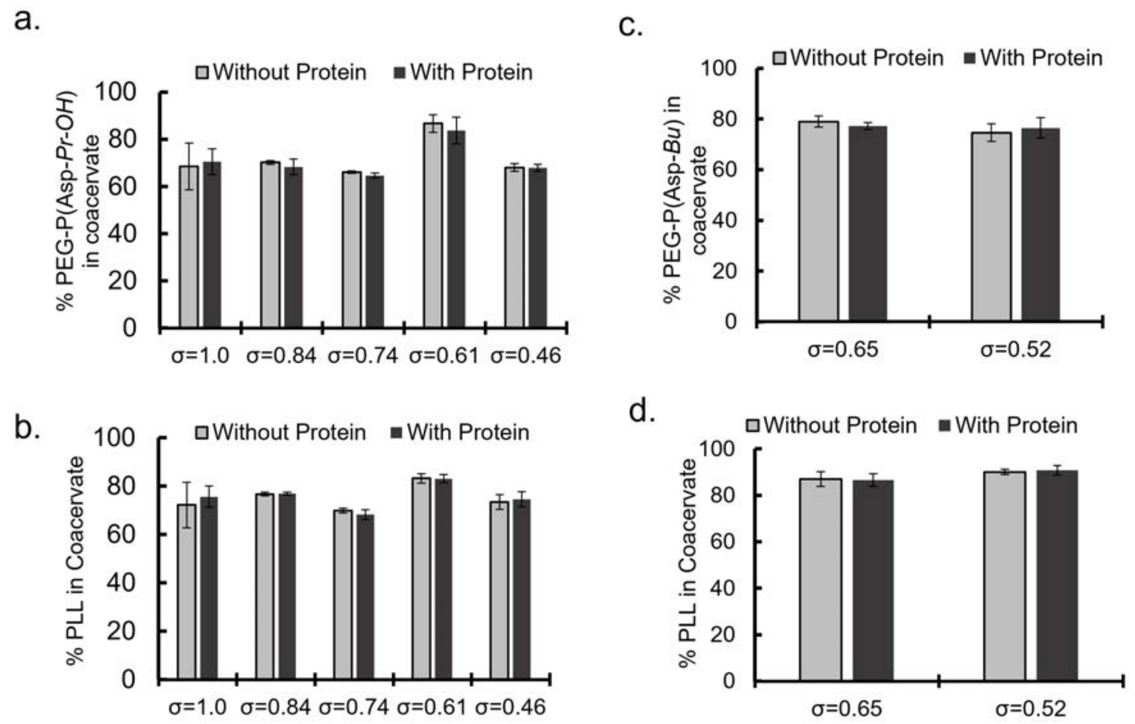

Fig. S22. Remaining amounts (%) of PLL and modified PEG-PAsp with different σ in the coacervate. (a, b) For PEG-P(Asp-*Pr-OH*)-based coacervates, and (c, d) for PEG-P(Asp-*Bu*)-based coacervates.

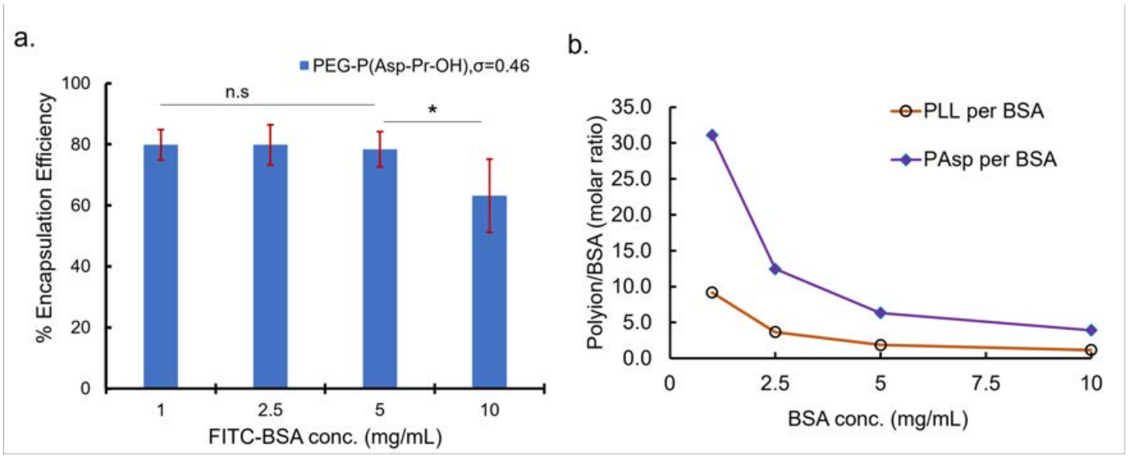

Fig. S23. a. Concentration-dependent change in % encapsulation efficiency of BSA for the coacervate prepared from PEG-P(Asp-*Pr-OH*) with σ = 0.46. b. Polyion-to-protein ratio in the coacervate at different BSA concentrations.

Please do not adjust margins

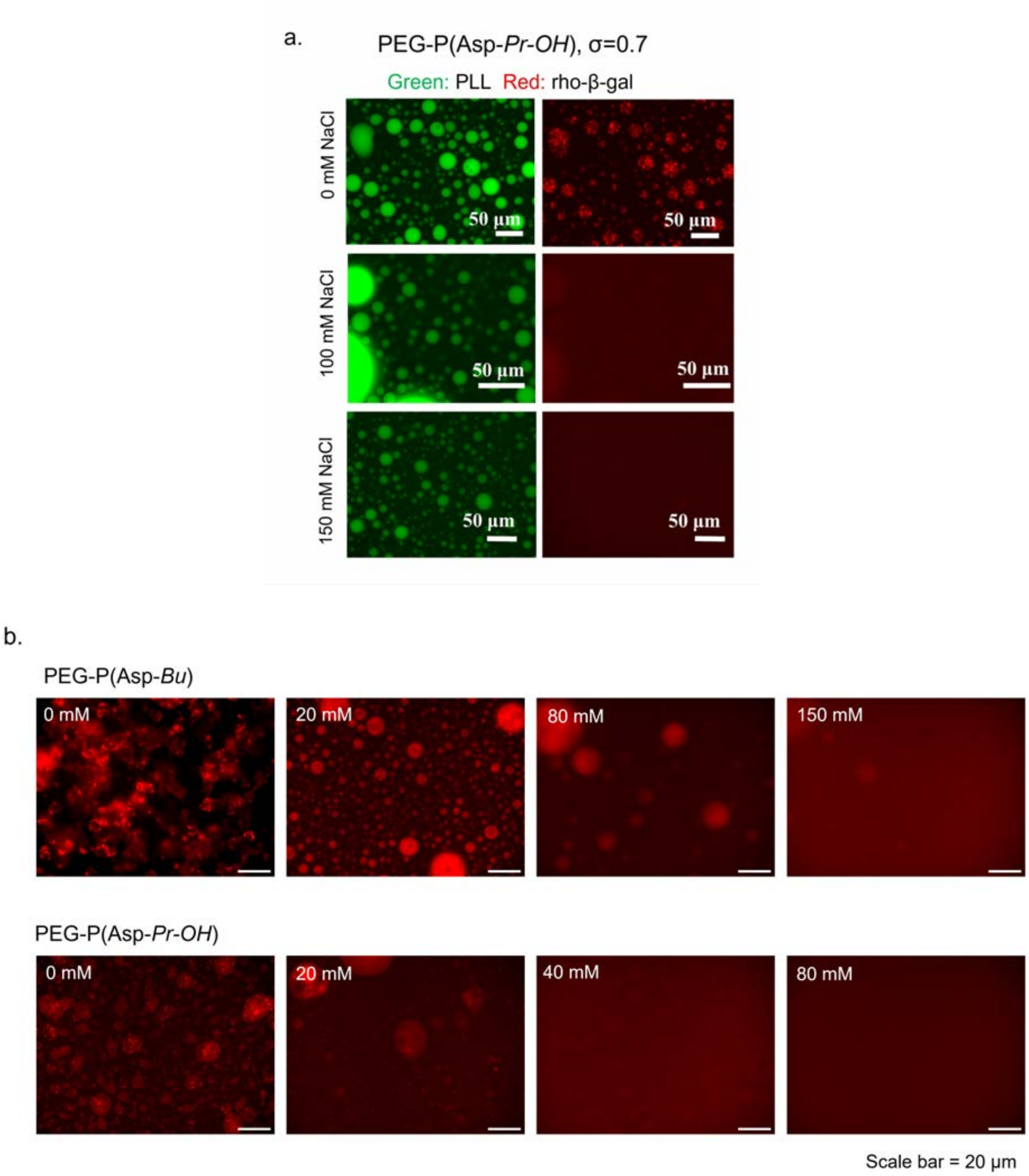

Fig. S24. a. Fluorescence images of the complex coacervates prepared from FITC-PLL (green), PEG-P(Asp-*Pr-OH*) with σ = 0.7, and rhodamine labelled β-gal (red) at different NaCl concentrations. b. Effect of NaCl concentrations after protein (rhodamine labelled β-gal, red) loading into coacervates prepared from PEG-P(Asp-*Bu*) or PEG-P(Asp-*Pr-OH*) with σ = 0.7.

 This journal is © The Royal Society of Chemistry 20xx

Please do not adjust margins

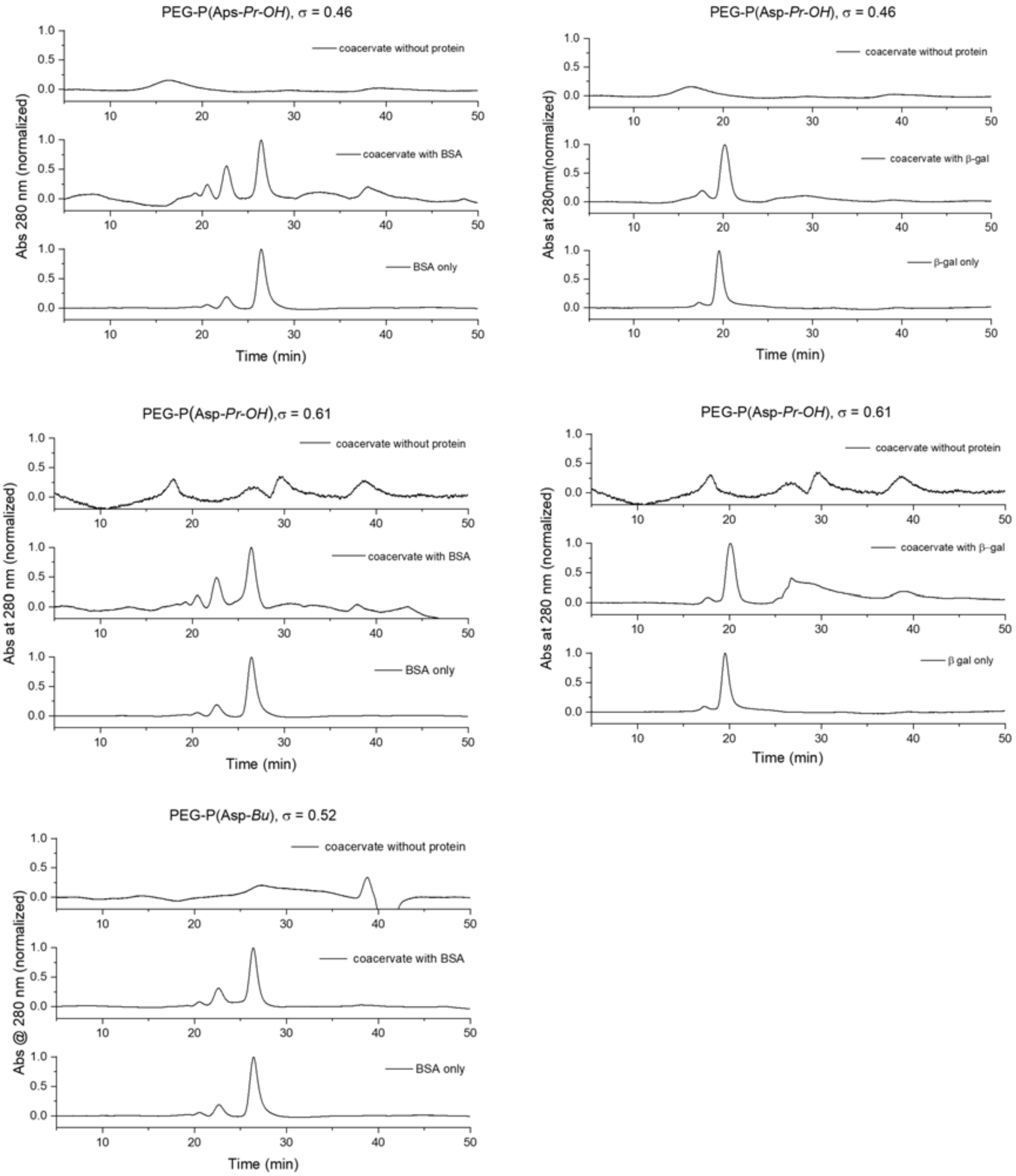

Fig. S25. Size exclusion chromatograms of the various coacervates from polyanions with different σ after the treatment with high-ionic-strength buffer (500 mM NaCl, 10 mM PB). The chromatograms for the coacervates with or without loading proteins were compared with that of the pure protein.

Please do not adjust margins

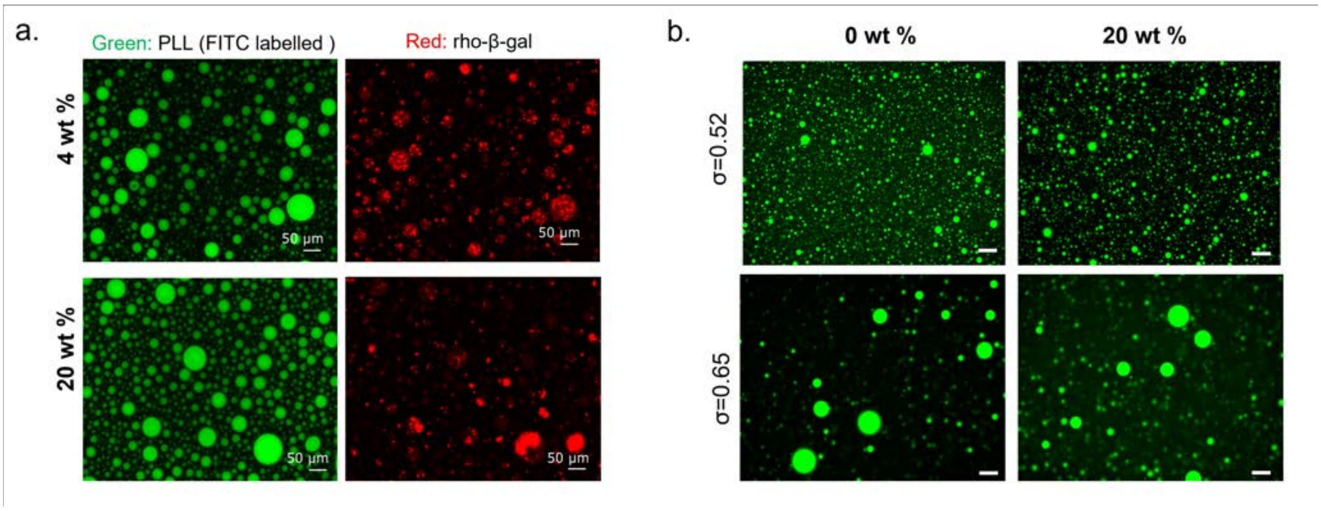

Fig. S26. Fluorescence images of coacervate droplets encapsulating proteins after the hexane-1,6-diol treatment with different conditions. a. Coacervates were prepared from PEG-P(Asp-*Pr-OH*) with σ = 0.61, where Rhodamine-β-gal (rho-β-gal, red) was loaded, were treated with 4 and 20 wt% hexane-1,6-diol. b. Coacervates were prepared from PEG-P(Asp-*Bu*) with σ=0.52 and 0.65, where FITC-BSA (green) was loaded, were treated with 0 and 20 wt% hexane-1,6-diol. Scale bars: 50 µm

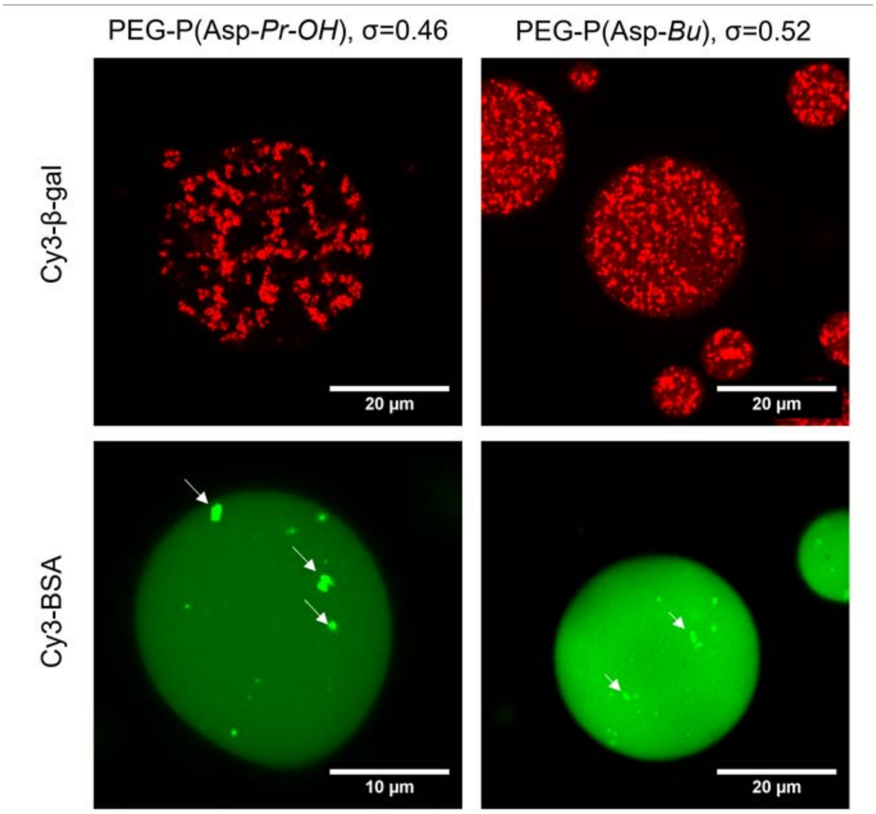

Fig. S27. A typical image of protein agglomeration within the PEG-P(Asp-*Pr-OH*), σ=0.46 and PEG-P(Asp-*Bu*)-based coacervate, σ=0.52 encapsulating Cy3-β-gal (red) and Cy3-BSA (green, false colour for clarity) incubated overnight at 4 °C. White arrows showed the agglomerated granules within the liquid coacervate.

This journal is © The Royal Society of Chemistry 20xx

Please do not adjust margins

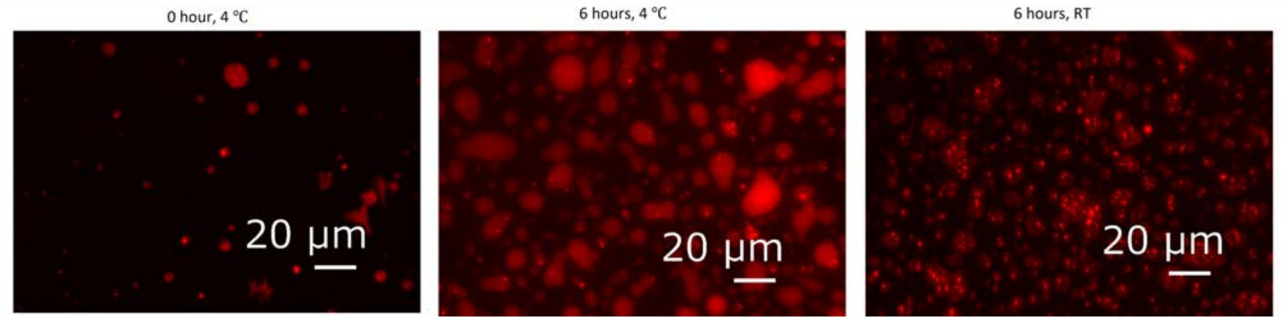

Figure S28. Fluorescence images of PEG-P(Asp-*Pr-OH*)-based coacervates encapsulating rho-β-gal (red) just after preparation at 4 °C (left), after 6-h equilibration at 4 °C (middle), and 6-h equilibration at room temperature (right). The increase of bright spots in the droplets indicates agglomeration of rho-β-gal.

Table S3: Different proteins used for encapsulation study and their information on total number of charges and molecular weights (MW).

Proteins		PDB ID	Total Negative Charges (Asp and Glu)	Total Positive Charges (Lys and Arg)	Total number of Charges	Net Charges	MW (KDa)
BSA		3V03	99	82	181	-17	66.5
β-gal (one unit of tetramer)		1JYV	126	86	212	-40	116
EGFP		4KEX	35	26	61	-9	28.6
Hen egg white Lysozyme		2EPE	9	17	26	+8	14.3
Ofatumumab (IgG)	Fab region	3GIZ	33	38	71	+5	150*
	Fc region	1H3X	22	25	47	+3	
Glucose Oxidase (GOx)		1GAL	66	37	103	-29	160
Horse Radish Peroxidase (HRP)		1W4W	31	27	58	-4	44
Diamine Oxidase (DAO)		3HI7	75	69	144	-6	170

* Sum of Fab region and Fc region

Table S4: Comparison of charge/mass ratio (e/kDa) for different proteins and polyions used in this study.

**Proteins**	**e/kDa**	**Polyions**	**e/kDa**
BSA	-0.26	PEG-PAsp, σ=1.0	-7.1
β-gal (one unit of tetramer)	-0.34	PEG-P(Asp-*Pr-OH*)	
EGFP	-0.31	σ=0.84	-5.6
Hen egg white Lysozyme	0.56	σ=0.74	-4.7
Ofatumumab (IgG)	0.05	σ=0.69	-4.3
Glucose Oxidase (GOx)	-0.18	σ=0.61	-3.6
Horse Radish Peroxidase (HRP)	-0.09	σ=0.46	-2.7
Diamine Oxidase (DAO)	-0.04	PEG-P(Asp-*Bu*)	
GFP(+33)	1.13	σ=0.87	-5.8
GFP(-29)	-1.02	σ=0.70	-4.4
		σ=0.65	-4.0
		σ=0.52	-3.1
		PLL-HBr	7.7

Please do not adjust margins

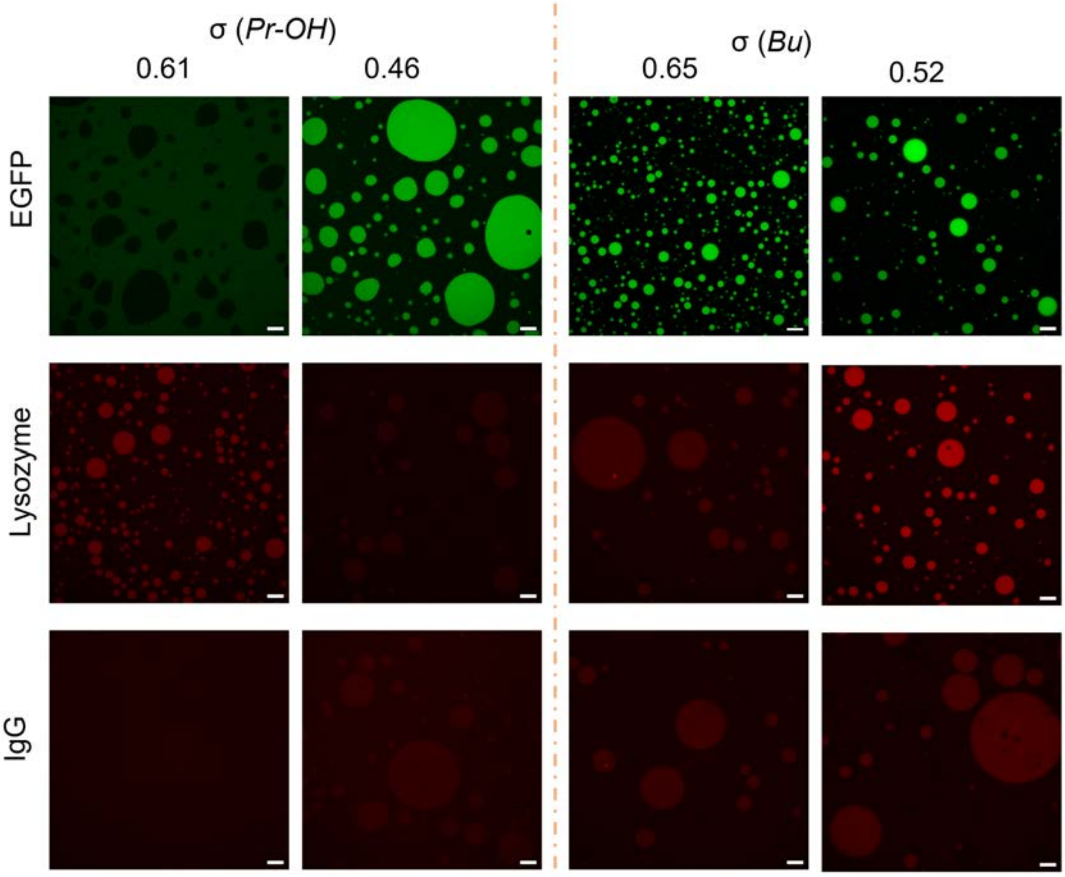

Fig. S29. Fluorescence images of charge-reduced-polymer-based complex coacervate droplets prepared in the presence of proteins (EGFP (green), Lysozyme (rhodamine labelled, red) and IgG (rhodamine labelled, red)).

This journal is © The Royal Society of Chemistry 20xx

Please do not adjust margins

Please do not adjust margins

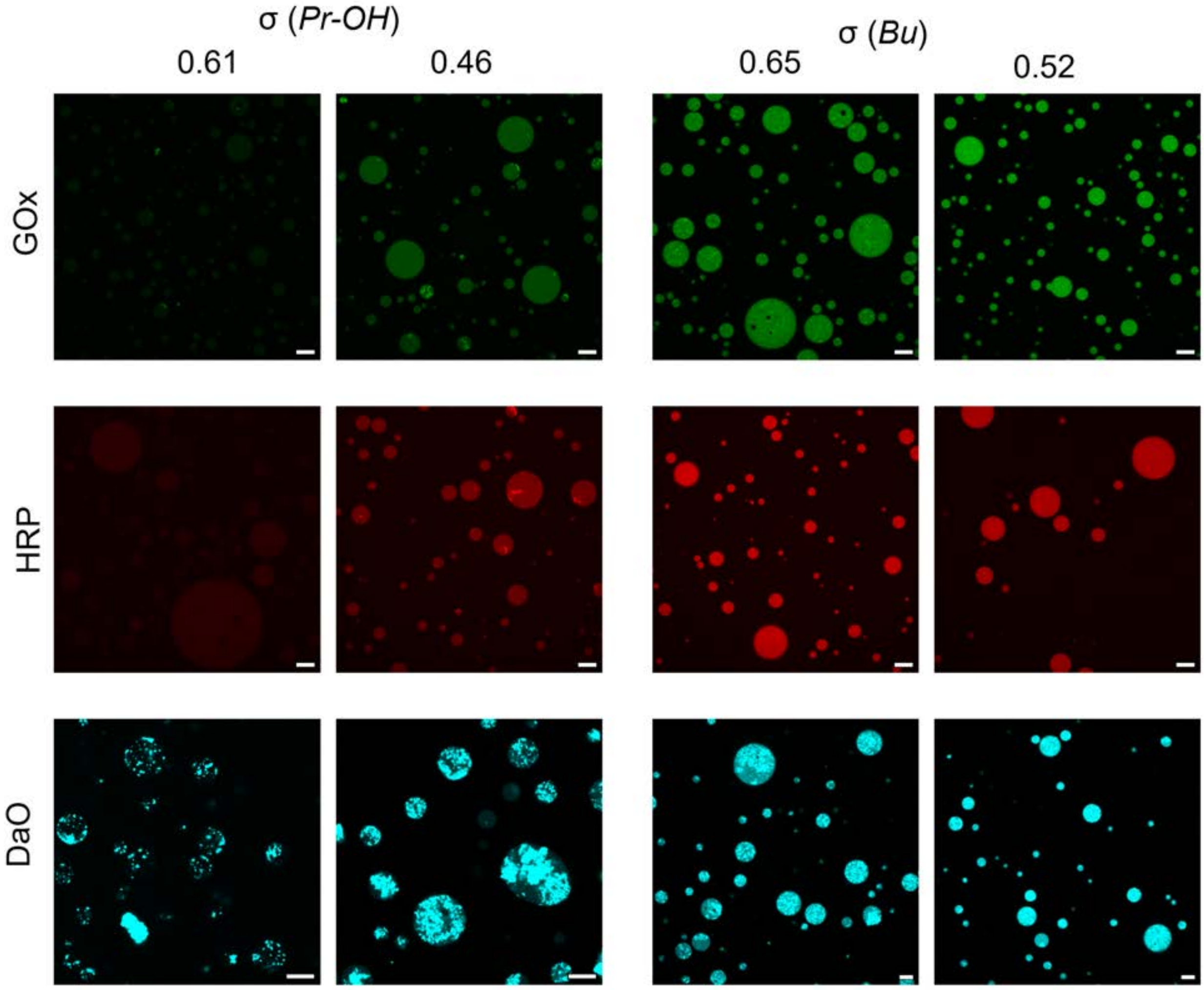

Fig. S30. Fluorescence images prepared from charge-reduced-polymer-based complex coacervate droplets in the presence of proteins (Gox (FITC labelled, green), HRP (rhodamine labelled, red) and DaO (cy5 labelled, cyan))

Please do not adjust margins

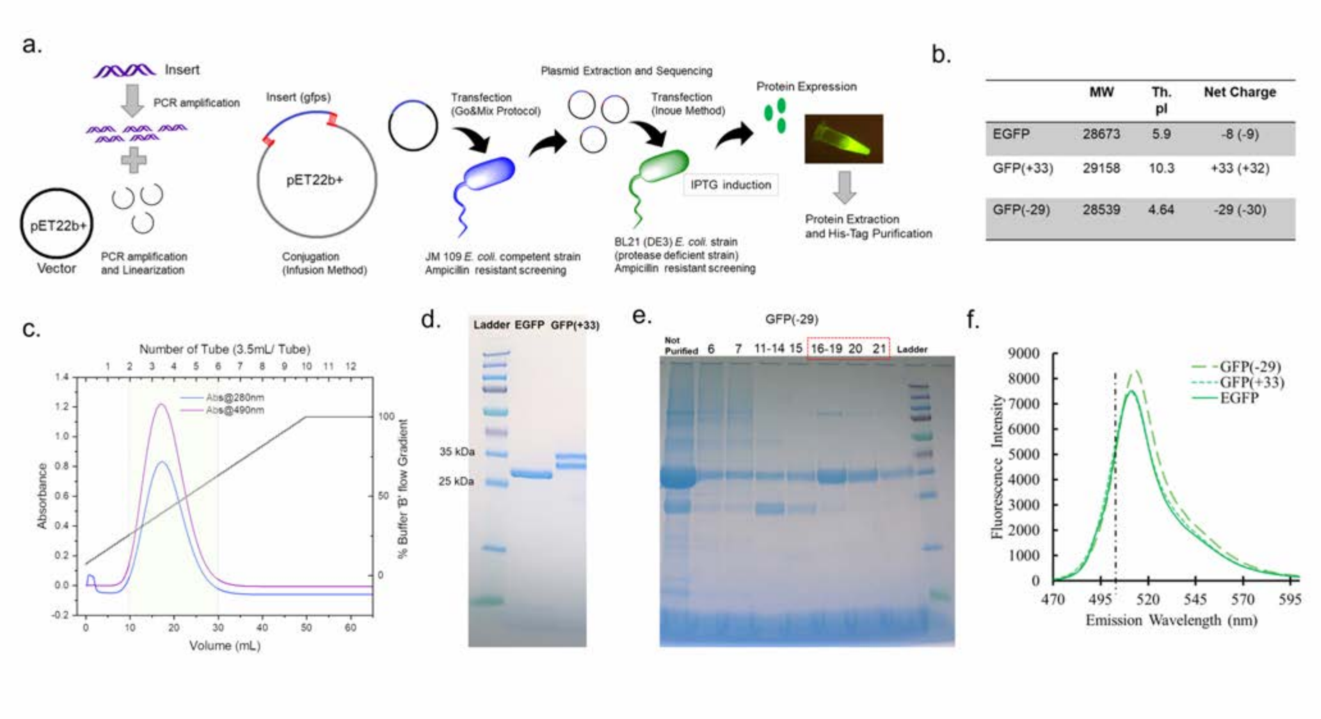

Fig. S31. Preparation of GFP isoforms. a. Schematic flow of the protein expression in E. coli. b. Information on GFP isoforms. Molecular weight (MW), theoretical p*I* (Th. p*I*), and net charges. c. His-Tag column elution profiles for EGFP with the buffer B (20 mM Tris HCl, 0.5 M NaCl, 0.5 M imidazole, pH 7.4) d. Coomassie-blue stained SDS-PAGE for EGFP and GFP (+33) e. Coomassie-blue stained SD-PAGE of different fraction of GFP (-29) after size-exclusion chromatography purification (only fractions 16-21 were collected.) f. Native PAGE of the different isoforms of GFPs. GFP (-29) moved further compared to EGFP. No band in GFP (+33) was confirmed due to opposite direction of mobility in the gel g. Emission spectra of GFP isoforms with excitation at 488 nm.

 This journal is © The Royal Society of Chemistry 20xx

Please do not adjust margins

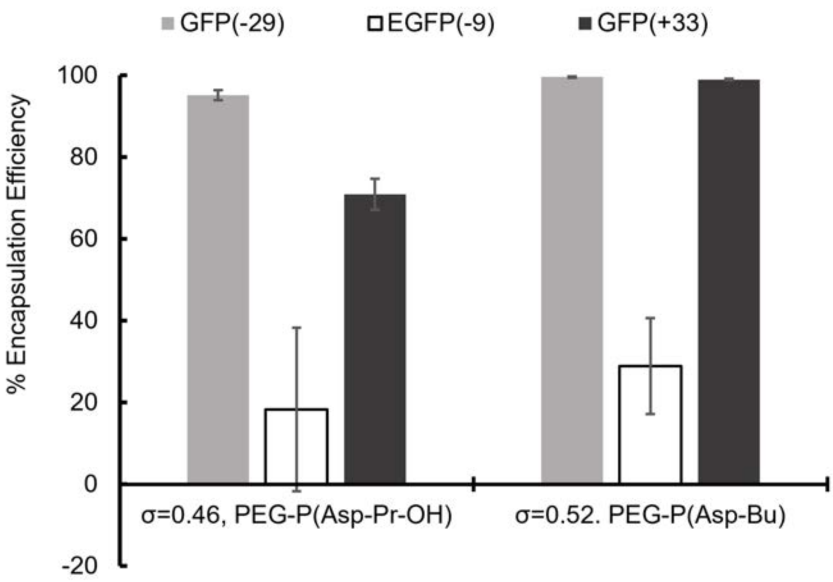

Fig. S32. Encapsulation efficiency (%) of charge-reduced-polymer-based coacervates for three isoforms of GFPs.

Please do not adjust margins

Please do not adjust margins

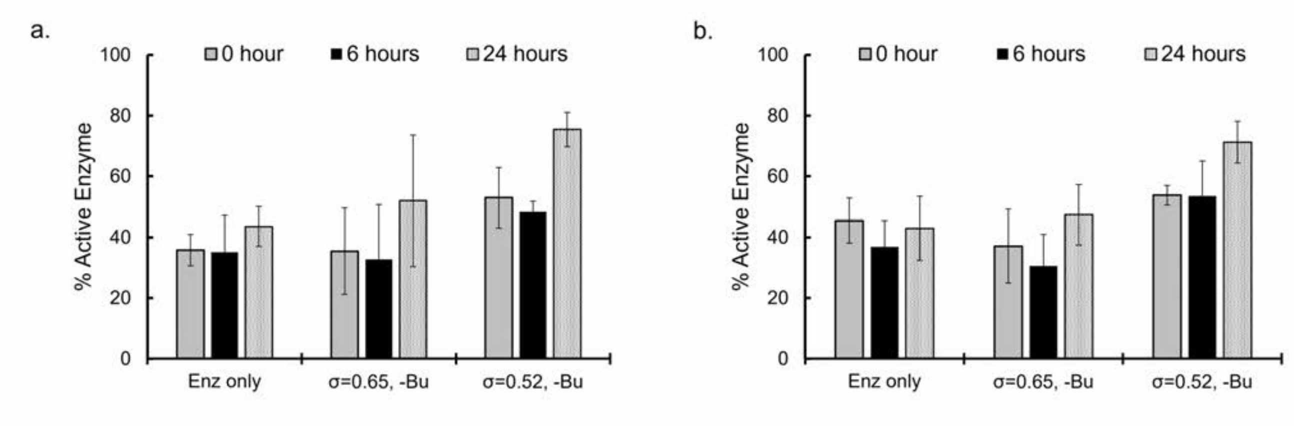

Fig. S33. Active enzyme fraction (%) recovered from the PEG-P(Asp-Bu)-based coacervates when stored at a. 4 °C and b. room temperature for glucose oxidase.

 This journal is © The Royal Society of Chemistry 20xx

Please do not adjust margins

Please do not adjust margins

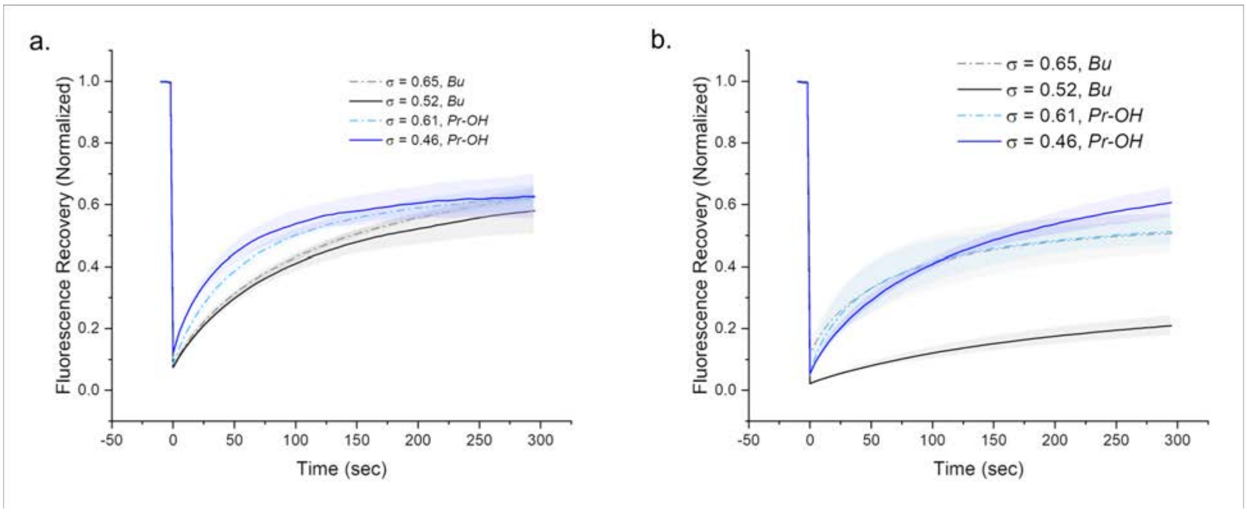

Fig. S34. FRAP results for Cy3-labelled PLL using various charge-density-reduced coacervates. a. Without protein, and b. with protein (BSA). PLL mobility reduced significantly at higher butyl modification in the presence of protein. Smear colour region indicate ±S.D.

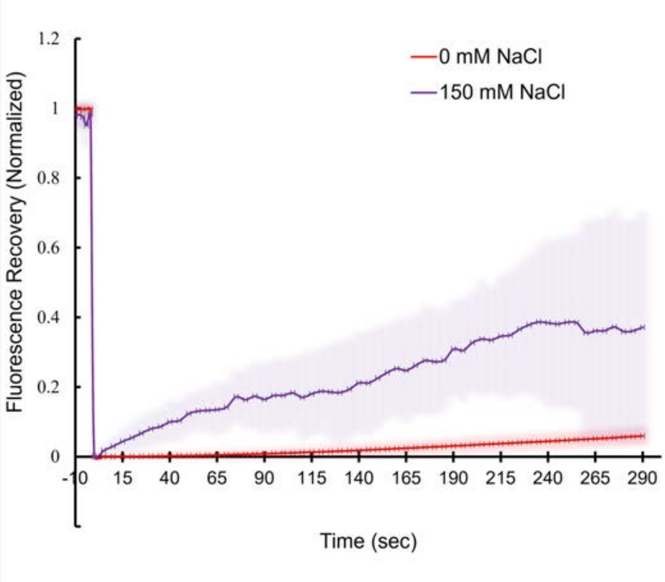

Fig. S35. FRAP results of Cy3-BSA loaded PEG-P(Asp-*Bu*)-based coacervate (σ=0.52) prepared at different NaCl concentrations. Mobility of Cy3-BSA increased at 150 mM NaCl compared to that at 0 mM NaCl. Smear colour region indicate ±S.D

Please do not adjust margins

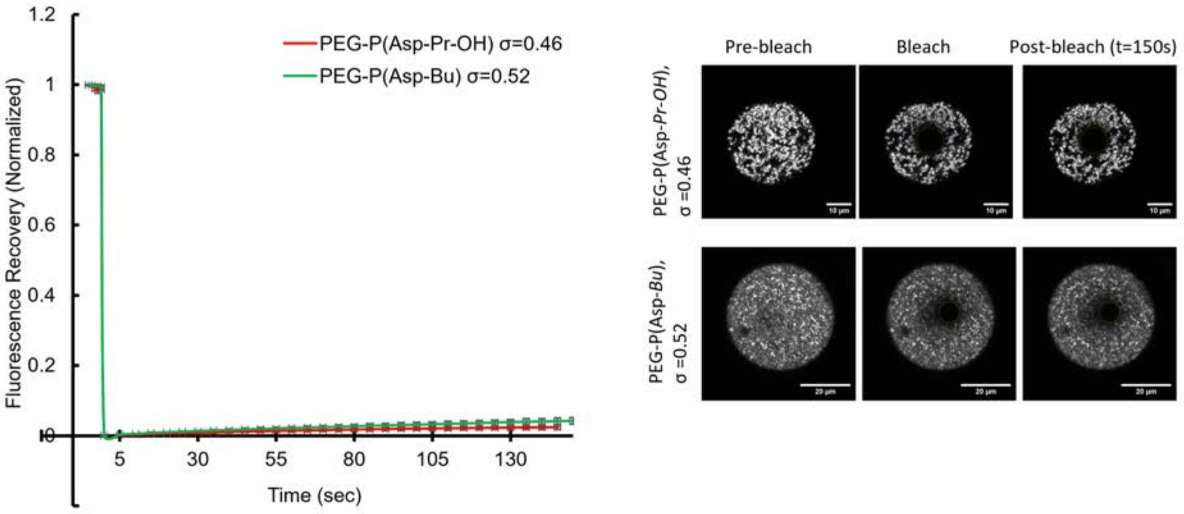

Fig. S36. FRAP results of Cy3-labelled β-gal loaded in coacervates prepared from PEG-P(Asp-Pr-OH), σ=0.46, and PEG-P(Asp-Bu), σ=0.52.

Movie S1 (.avi). Time-lapsed images of sequestration of rho-β-gal (red) into the coacervate prepared from PEG-P(Asp-*Pr-OH*), σ=0.46.

Movie S2(.avi). Time-lapsed images of sequestration of FITC-BSA (green) into the coacervate prepared from PEG-P(Asp-*Pr-OH*), σ=0.46.

Movie S3 (.mp4). Sequestration of rho-β-gal (red) into the FITC-BSA (green) pre-loaded coacervate prepared from PEG-P(Asp-*Pr-OH*), σ=0.46.

6. References:

1 T. Nojima and T. Iyoda, Water-Rich Fluid Material Containing Orderly Condensed Proteins, *Angewandte Chem International Edition*, 2017, **56**, 1308-1312.
2 P. N. O. Kasimbeg, F. C. Cheong, D. B. Ruffner, J. M. Blusewicz and L. A. Philips, Holographic Characterization of Protein Aggregates in the Presence of Silicone Oil and Surfactants, J Pharm Sci, 2019, **108**, 155-161.
3 M. A. Odete, F. C. Cheong, A. Winters, J. J. Elliott, L. A. Philips and D. G. Grier, The role of the medium in the effective-sphere interpretation of holographic particle characterization data, *Soft matter*, 2019.
4 A. Winters, F. C. Cheong, M. A. Odete, J. Lumer, D. B. Ruffner, K. I. Mishra, D. G. Grier and L. A. Philips, Quantitative Differentiation of Protein Aggregates From Other Subvisible Particles in Viscous Mixtures Through Holographic Characterization, *Journal of Pharmaceutical Sciences*, 2020, **109**, 2405-2412.
5 M. Nakanishi, J.-S. Park, W.-D. Jang, M. Oba and K. Kataoka, Study of the quantitative aminolysis reaction of poly(β-benzyl l-aspartate) (PBLA) as a platform polymer for functionality materials, *Reactive and Functional Polymers*, 2007, **67**, 1361-1372.
6 Y. Liu, T. Maruyama, B. KC, T. Mori, Y. Katayama and A. Kishimura, Inducible Dynamic Behavior of Polyion Complex Vesicles by Disrupting Charge Balance, *Chemistry Letters*, 2021, **50**, 1034-1037.
7 T. Ohta, T. Norisuye, A. Teramoto and H. Fujita, Solution Properties of Synthetic Polypeptides. XX. Light-Scattering Study of Poly-N5-(2-hydroxyethyl)L-glutamine in the Helix—Coil Transition Region, *Polymer Journal*, 1976, **8**, 281-287.
8 H. Inoue, H. Nojima and H. Okayama, High efficiency transformation of Escherichia coli with plasmids, *Gene*, 1990, **96**, 23-28.
9 D. B. Thompson, J. J. Cronican and D. R. Liu, Engineering and identifying supercharged proteins for macromolecule delivery into mammalian cells, *Methods Enzymol*, 2012, **503**, 293-319.
